# Nanofiber Composite Reinforced Organohydrogels for Multifunctional and Wearable Electronics

**DOI:** 10.1007/s40820-023-01148-9

**Published:** 2023-07-07

**Authors:** Jing Wen, Yongchuan Wu, Yuxin Gao, Qin Su, Yuntao Liu, Haidi Wu, Hechuan Zhang, Zhanqi Liu, Hang Yao, Xuewu Huang, Longcheng Tang, Yongqian Shi, Pingan Song, Huaiguo Xue, Jiefeng Gao

**Affiliations:** 1 School of Chemistry and Chemical Engineering, Yangzhou University, Yangzhou, 225002 People’s Republic of China; 2 Testing Center, Yangzhou University, Yangzhou, 225002 People’s Republic of China; 3 Key Laboratory of Organosilicon Chemistry and Material Technology of Ministry of Education, Hangzhou Normal University, Hangzhou, 311121 People’s Republic of China; 4 College of Environment and Safety Engineering, Fuzhou University, Fuzhou, 350116 People’s Republic of China; 5 Centre for Future Materials, University of Southern Queensland, Springfield Central, 4300 Australia

**Keywords:** Composite organohydrogel, Multi-level interfacial bonding, Mechanical properties, Strain sensor, Electromagnetic interference shielding

## Abstract

Composite organohydrogels have been widely used in wearable electronics. However, it remains a great challenge to develop mechanically robust and multifunctional composite organohydrogels with good dispersion of nanofillers and strong interfacial interactions. Here, multifunctional nanofiber composite reinforced organohydrogels (NCROs) are prepared. The NCRO with a sandwich-like structure possesses excellent multi-level interfacial bonding. Simultaneously, the synergistic strengthening and toughening mechanism at three different length scales endow the NCRO with outstanding mechanical properties with a tensile strength (up to 7.38 ± 0.24 MPa), fracture strain (up to 941 ± 17%), toughness (up to 31.59 ± 1.53 MJ m^−3^) and fracture energy (up to 5.41 ± 0.63 kJ m^−2^). Moreover, the NCRO can be used for high performance electromagnetic interference shielding and strain sensing due to its high conductivity and excellent environmental tolerance such as anti-freezing performance. Remarkably, owing to the organohydrogel stabilized conductive network, the NCRO exhibits superior long-term sensing stability and durability compared to the nanofiber composite itself. This work provides new ideas for the design of high-strength, tough, stretchable, anti-freezing and conductive organohydrogels with potential applications in multifunctional and wearable electronics.

**Supplementary Information:**

The online version contains supplementary material available at 10.1007/s40820-023-01148-9.

## Highlights

- A nanofiber composite reinforced organohydrogel with multifunctionality is prepared.
- The composite organohydrogel possesses multiple interfacial bondings and multi-level strengthening and toughening mechanism is proposed.
- The composite organohydrogel exhibits long-term strain sensing stability and can be used for high performance electromagnetic interference shielding.

**Supplementary Information:**

The online version contains supplementary material available at 10.1007/s40820-023-01148-9.

## Graphical abstract

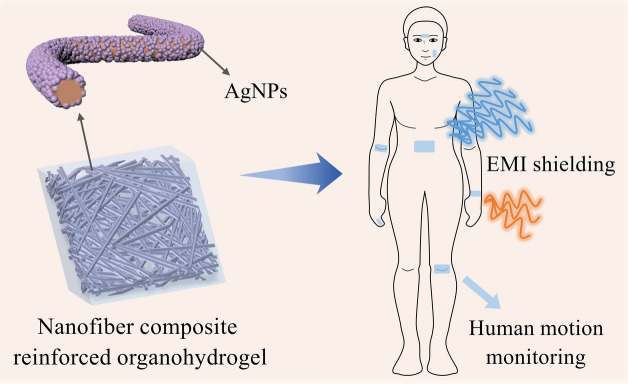

## Introduction

Organohydrogels with excellent flexibility, stretchability, biocompatibility and freezing resistance have received great interest and have been good candidates for wearable electronics [1–4]. Generally, organohydrogels are composed of a crosslinked polymer network in a mixture of water and organic solvent [5, 6]. Two strategies, namely chemical and physical crosslinking, are often used for fabricating organohydrogels [7–9]. The chemical crosslinking is usually completed during the polymerization [10]. On the other hand, physically crosslinked organohydrogels are usually prepared by cyclic freezing and thawing or the solvent exchange. The freezing and poor solvent can induce the macromolecular chain aggregation and further gelation, and polymer network is constructed when sufficient crosslinked points are formed [11–14].

Due to the intrinsically high liquid content as well as loose polymeric structure, organohydrogels often show weak mechanical performance, although their mechanical properties could be tuned by adjusting the ratio between the organic solvent and water [15, 16]. Also, the organohydrogels usually lack the functionality, severely limiting their applications. Thus, nanofillers are incorporated into the organohydrogels to improve the mechanical properties as well as the functionality [17–19]. For instance, cellulose nanofibrils (CNFs) and metal salt were used to enhance the polyvinyl alcohol (PVA) organohydrogels and ionic conductivity. The elongation at break and toughness of the obtained ionic organohydrogel could reach as high as 660% and 5.25 MJ m^−3^, respectively, and the ionic conductivity endowed the organohydrogel with strain sensing performance [8]. In addition to the ionic organohydrogels, electrically conductive composite hydrogels were fabricated by using the carbon nanomaterials such as MXene and carbon nanotubes (CNTs) as nanofillers [5, 20, 21]. For example, polydopamine modified CNTs were mixed with the glycerol/water containing the copolymers, and the composite hydrogels with enhanced mechanical behavior and electrical conductivity were obtained after copolymerization. Liao et al. prepared MXene based organohydrogel through polymerization and solvent exchange, and the composite organohydrogel could be used for strain sensing and body motions monitoring [21].

Although the nanofillers may improve, to a certain degree, the mechanical and electrical properties of organohydrogels, they are easily aggregated especially at a high concentration. Furthermore, it is still difficult to achieve the good interfacial interaction between the nanofillers and macromolecules and hence fully transfer the stress from the soft polymer to rigid nanofillers. Therefore, the poor dispersion of nanofillers and weak interfacial interactions greatly limit the enhancement and even lead to the decline of the mechanical performance. Also, the functionality such as strain sensing may become unstable especially during the long-term use.

To solve these issues, we develop a nanofiber composite reinforced organohydrogel with a sandwich-like structure by combination of blading coating and freezing–thawing. The nanofiber network was pre-constructed, avoiding the nanofiber aggregation. In addition to enhancement of mechanical properties, the nanofiber composite with high electrical conductivity endows the organohydrogels with multifunctionality. NCROs possess excellent environmental tolerance such as anti-freezing performance, and can be used for high performance electromagnetic interference (EMI) shielding and strain sensing. Particularly, due to gel stabilized conductive network of the nanofiber composite membrane with a porous structure, the NCRO strain sensor exhibits outstanding cyclic and long-term sensing stability and durability, which cannot be achieved for the nanofiber composite itself. The “nanofiber composite reinforcement” method can provide inspiration for preparation of mechanically robust and multifunctional organohydrogels with promising applications in flexible electronics and people’s healthcare monitoring.

## Experimental Section

### Materials

Polyvinyl alcohol (PVA-1799, 98–99% hydrolyzed, Aladdin), glycerol (Aladdin), polyurethane (PU, BASF), polyvinyl pyrrolidone (PVP, M_w_ = 10,000, Aladdin), silver trifluoroacetate (STA, AgCF_3_COO, 99.1%, Shanghai Bide Pharmatech Co. Ltd.), absolute ethanol (Sinopharm Chemical Reagent Co. Ltd.), N,N-dimethylformamide (DMF, Sinopharm Chemical Reagent Co. Ltd.), tetrahydrofuran (THF, Shanghai Macklin Biochemical Co. Ltd.) and hydrazine hydrate (N_2_H_4_·H_2_O, 85%, Sinopharm Chemical Reagent Co. Ltd.) were used as supplied.

### Preparation of PVA/Glycerol/Water Solution

20 wt% PVA solution was prepared by dissolving PVA powders in deionized water under vigorous stirring in an oil bath at 100 °C for 5 h. After defoaming by standing at room temperature (~ 25 °C) for 5 h, a clear solution was obtained. Then, the PVA solution and glycerol were mixed with a weight ratio of 1:1, and subject to vigorous stirring for 30 min (100 °C). Finally, the transparent PVA/glycerol/water solution was obtained after defoaming at 60 °C for 2 h.

### Preparation of PVP/Ag@PU Nanofiber Composite Membranes

Firstly, PU pellets were dissolved in a solvent consisting of DMF and THF with a weight ratio of 4:1 and the above mixture was stirred at 65 °C for 12 h to obtain PU solution (15 wt%). The electrospinning process was completed at a humidity of 30–35%. The PU solution in a plastic syringe was pushed out through a metallic needle at a voltage of 15 kV and a feed rate of 1 mL h^−1^. The distance between the needle and the rotating drum collector covered with an aluminum foil was 12 cm. After removed from the collector, the aluminum foil was placed in an air-circulating oven at 60 °C for 6 h to remove the remaining solvent, and then the PU nanofiber membrane was obtained. Whereafter, the as-prepared membrane was submerged in the PVP/STA solution for 1 h (25 °C), which was prepared by dissolving PVP and STA powders in absolute ethanol (The PVP and STA concentration are 4 and 10 wt% respectively). After dried at 80 °C for 30 s, the membrane was soaked into the N_2_H_4_·H_2_O solution for 1 h (25 °C), where the Ag precursors were entirely converted into Ag nanoparticles. Finally, the obtained PVP/Ag@PU nanofiber composite membrane was rinsed with deionized water and then dried at 37 °C for 1 h. The obtained composite membrane is described as PVP-XAg@PU, where X stands for the proportion of STA in the PVP/STA solution.

### Fabrication of Composite Organohydrogels

A certain amount of PVA/glycerol/water solution was poured onto a glass sheet, and then a film applicator was used to flatten the solution on the glass sheet. After that, the nanofiber composite membrane was immediately deposited on the solution. Another part of PVA/glycerol/water solution was heated and then quickly poured onto the membrane on the glass sheet, and the upper solution was spread again with the film applicator. Finally, the nanofiber composite membrane interleaved solution was frozen at -25 °C for 12 h and thawed at 25 °C for 3 h.

### Mechanical Properties Tests

With a universal tensile machine (Instron Model 3367, USA), all mechanical tests on the composite organohydrogels were performed at room temperature. The composite organohydrogels were tailored into dumbbell-shaped samples (gauge length: 50 mm, width: 4 mm) by using a dumbbell-shaped cutter. The thickness of each sample was measured with a digital thickness gauge. The speed for the tensile tests was fixed at 50 mm min^−1^. Then, the stress of the composite organohydrogel sample was measured from the force divided by the initial cross-sectional area of the sample, and the strain (*ε*) was calculated by dividing the measuring distance (*L*_*m*_) with the initial distance (*L*_*0*_) of the sample (Eq. (1)):

1 $$\varepsilon = \frac{{L_{m} }}{{L_{0} }} \times 100\%$$

Tensile toughness is defined as the integrated area under the stress–strain curve from zero to the point of fracture.

### Pure Shear Tests

The fracture energy of the composite organohydrogels was assessed by using pure shear tests [22]. Two different rectangular samples (50 mm × 10 mm), notched and unnotched, were used to obtain force–displacement curves. The notched samples with a notch length of one-third of the width were prepared by using a razor blade. When the notch transforms into a running crack, the critical extension is designated as Δ*L*_*c*_, and the work required for an unnotched specimen to reach Δ*L*_*c*_ is designated as *U*(Δ*L*_*c*_). In addition, the sample’s cross section area is denoted as *A*. The fracture energy was calculated by Eq. (2):

2 $$\Gamma = \frac{{U\left( {\Delta L_{c} } \right)}}{A}$$

### SEM Characterization

The microstructures of the composite organohydrogels and the nanofiber composite membranes were characterized by a field emission scanning electron microscopy (FE-SEM, Zeiss Supra55, Germany) with the acceleration voltage of 5 kV. In order to completely replace the glycerol with water, all organohydrogel samples were immersed in deionized water for 24 h. Subsequently, the samples were fractured in liquid nitrogen and then freeze dried using a freeze drying device (SCIENTZ-10N freeze drier, China). Before the SEM observation, a layer of gold covered the fracture surfaces of the freeze-dried samples.

### Rheological Measurements

A DHR rheometer (TA, USA) was used for the rheological tests. The samples with a diameter of 25 mm were used to measure the storage modulus (*G'*) and loss modulus (*G"*). The oscillation strain sweep test was carried out at a constant frequency of ω = 6.28 rad s^−1^ with a strain sweep from 0.01% to 100%. The time and temperature sweep tests (ω = 6.28 rad s^−1^ and γ = 0.1%) were performed with a time range of 0–200 s and a temperature range of 25–60 °C, respectively.

### Stress Relaxation Tests

In the stress relaxation tests, the dumbbell-shaped samples (50 mm × 4 mm) were stretched to 100% strain and then held at this strain point for 10 min. Meanwhile, the stress proportional to time was measured by the force sensor. When the strain reaches 100%, this moment is defined as time origin (t = 0), and the stress at this point is σ_0_. The variations of the stress ratio defined as σ_t_/σ_0_ showed the degree of stress relaxation in different samples.

### Small Angle X-ray Scattering (SAXS)

In the SAXS characterization (NanoSTAR, Bruker AXS, Germany), the length of the organohydrogel samples was 20 mm and the width was 6 mm. In the in-situ SAXS tests of the organohydrogels during the stretching process, the machine equipped with a stretcher served to stretch the samples to a fixed strain.

### Conductivity Tests

For the conductivity test of the composite organohydrogel, the resistance (*R*) of the sample was first measured by an electrometer (KEYSIGHT 6517B, USA). Then, the conductivity (*σ*) of the sample was calculated by Eq. (3):

3 $$\sigma { = }\frac{L}{RA}$$

where *L* is the length of the sample and *A* is the cross-sectional area of the sample.

### Strain Sensing Tests

To evaluate the sensing performances of the composite organohydrogels, the PVP/Ag@PU nanofiber composite membranes (50 mm × 10 mm) were first connected to two copper wires by using the silver paste adhesive which was then cured in an oven at 60 °C for 6 h. The strain sensing tests of the composite organohydrogels with the copper wires as the electrodes were performed on an electronic universal tensile machine (ZQ-990B-200, ZHIQU Precision Instrument Co. Ltd., China) with a stretching rate of 20 mm min^−1^. As different strains were applied to the samples, the transient resistance of the samples was recorded by an electrometer (KEYSIGHT 6517B, USA). For human motion detections, the sample was adhered on the skin surface of a volunteer to detect instantaneous resistance. The resistance with an applied strain is defined as *R*, and the original resistance is defined as *R*_0_. The relative resistance variation was calculated by Eq. (4):

4 $$\frac{\Delta R}{{R_{0} }}{ = }\frac{{R{ - }R_{0} }}{{R_{0} }}$$

The gauge factor (GF) was used to evaluate the strain sensitivity, and is given by Eq. (5):

5 $${\text{GF = }}\frac{{\Delta R{/}R_{0} }}{\varepsilon }$$

where *ε* is the strain of the composite organohydrogel.

### Electromagnetic Interference Shielding Measurements

The EMI shielding effectiveness (SE) values of the composite organohydrogels and the nanofiber composite membranes were measured by a vector network analyzer (Agilent N5230, USA) in the frequency of 8.2–12.4 GHz (X-band). The scattering parameters S_11_ and S_21_ were obtained from the analytical results of the rectangular samples with effective areas of 25 mm × 15 mm. The power coefficient of reflectivity (*R*), transmissivity (*T*) and absorptivity (*A*) were calculated by Eqs. (6–8) [23]:

6 $${\text{R = }}\left| {{\text{S}}_{{{11}}} } \right|^{{2}}$$

7 $${\text{T = }}\left| {{\text{S}}_{{{21}}} } \right|^{{2}}$$

8 $${\text{A + R + T = 1}}$$

Then, the reflection EMI shielding effectiveness (SE_R_) and the absorption EMI shielding effectiveness (SE_A_) could be described as [23]:

9 $${\text{SE}}_{{\text{R}}} { = }{ - }{\text{10log}}\left( {\text{1 - R}} \right)$$

10 $${\text{SE}}_{{\text{A}}} { = }{ - }{\text{10log}}\frac{{\text{T}}}{{\text{1 - R}}}$$

The total EMI shielding effectiveness (SE_T_) can be considered to be the sum of SE_A_, SE_R_ and SE_M_ [24]:

11 $${\text{SE}}_{{\text{T}}} {\text{ = SE}}_{{\text{A}}} {\text{ + SE}}_{{\text{R}}} {\text{ + SE}}_{{\text{M}}}$$

where SE_M_ is the multireflection EMI shielding effectiveness. When SE_T_ is higher than 10 dB, SE_M_ can be ignored. Hence, SE_T_ is given by Eq. (12):

12 $${\text{SE}}_{{\text{T}}} {\text{ = SE}}_{{\text{A}}} {\text{ + SE}}_{{\text{R}}} {\text{ = - 10logT}}$$

## Results and Discussion

### Design and Synthesis of the Nanofiber Composite Reinforced Organohydrogels (NCROs)

**Fig. 1:**
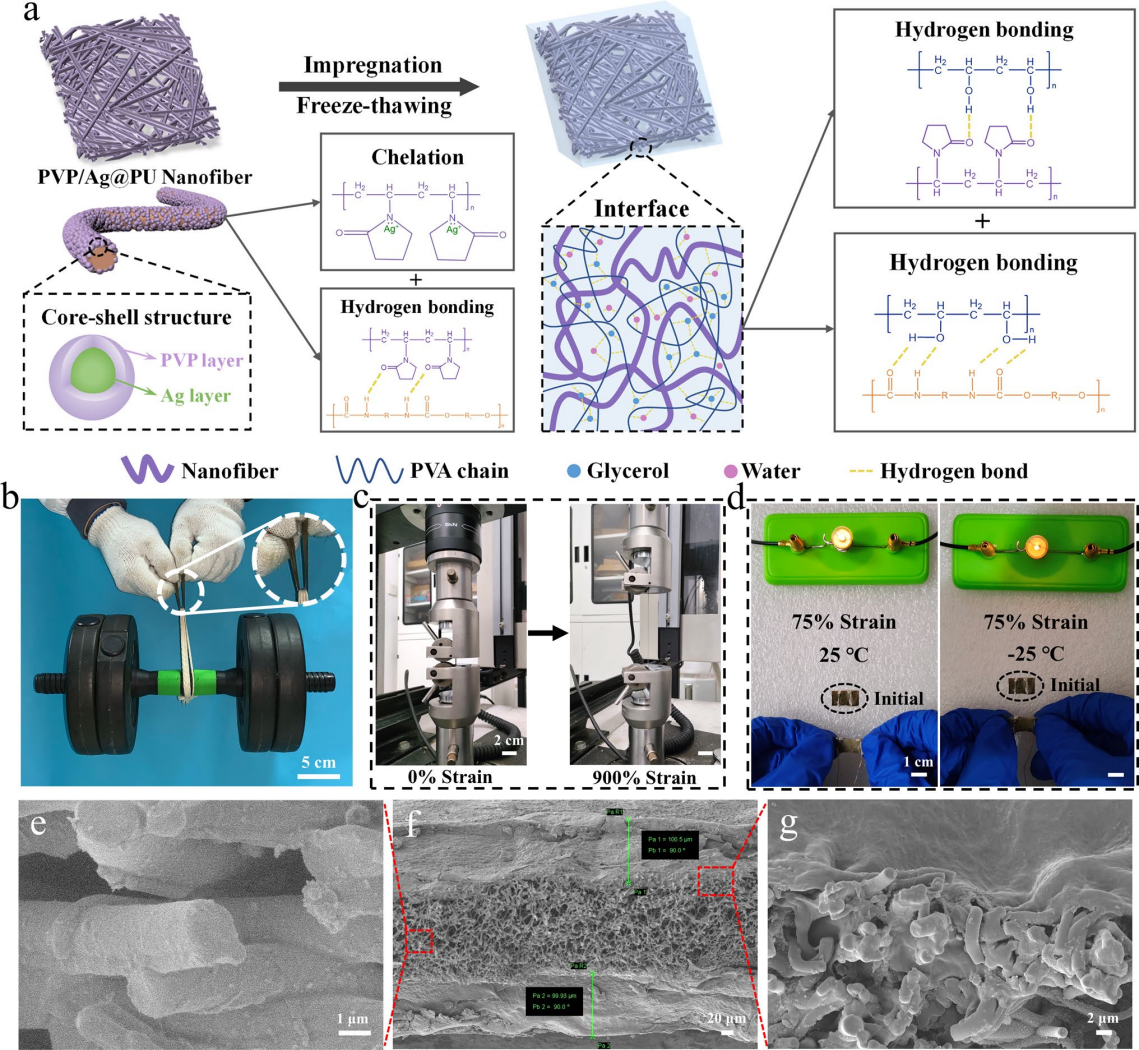
Preparation, microstructure and properties of the NCRO. **a** The schematic demonstration for the preparation and interfacial interactions of the composite organohydrogel. **b** The image of the NCRO maintaining a dumbbell (5 kg) with more than 7000 times its own weight. **c** Photographs of the NCRO being stretched. **d** Photos of the NCRO connected in circuits upon being stretched to 75% strain at 25 °C and − 25 °C, respectively. **e–g** Cross sectional SEM images of **f** the nanofiber composite reinforced organohydrogel with a sandwich-like structure, **e** the middle nanofiber composites layer and **g** the interfacial region between the nanofiber composite and top gel layer

A high-strength, anti-freezing and conductive composite organohydrogel was prepared via a nanofiber composite reinforcement method constructing multiple non-covalent interactions, which is schematically demonstrated in Fig. 1a. Firstly, the PVP/Ag@PU nanofiber composite membrane was obtained by combination of dip coating and ion reduction. In the step of dipping, the chelation between PVP and Ag^+^ due to electrostatic attraction [25] and the ion-dipole effect [26, 27] of trifluoroacetate anions (CF_3_COO^−^) and the hydroxyl groups (-OH) in ethanol promote the combination of Ag precursors and polyurethane (PU). Then, the membrane with Ag precursors was chemically reduced to Ag nanoparticles (AgNPs). In the PVP/Ag@PU nanofiber composite membrane, the multi-layer core-shell structure was formed. Next, the PVA/glycerol/water solution was evenly coated on both sides of the as-prepared composite membrane. Finally, the NCRO with a sandwich-like structure was prepared by using a freezing-thawing strategy, in which macromolecular chains aggregate to form a polymeric network, while glycerol also promoted the formation of strong polymer-solvent-polymer hydrogen bonds [15, 28]. Due to the mechanically interlocked network structure, the introduction of the composite membrane can significantly improve the mechanical properties of the organohydrogel. As shown in Fig. 1b, c, the NCRO exhibited high strength and excellent stretchability. In addition, the glycerol contained in the gel layers and AgNPs rich in the composite membrane endow the composite organohydrogel with anti-freezing properties and conductivity, respectively. It could be found from Fig. 1d that with the sample stretched to 75% strain, it could still light the small bulb in the circuit after freezing at − 25 °C for 1 h. Therefore, the electrically conductive NCRO can work normally in low temperature scenarios.

The conductivity of the composite organohydrogels was mainly determined by the Ag precursor concentration. As shown in Fig. S1, the conductivity of the composite organohydrogel reached 110.9 ± 1.2 S cm^−1^ at 10 wt% (Ag precursor concentration), which was more than 4 times that of 5 wt%. It could be found from Fig. S2a, b that due to the low Ag concentration of 5 wt%, only a small quantity of AgNPs were attached to the PU nanofibers and the AgNPs were small in the size, which greatly reduced the conductivity of the composite organohydrogel. To our satisfaction, AgNPs were uniformly distributed on the nanofibers with 10 wt% Ag concentration and the composite membrane possessed a complete and continuous conductive network (Fig. S2c, d). As exhibited in Fig. S3, the average size of AgNPs in the composite membrane (10 wt% Ag concentration) was around 152.72 nm. From the photograph in Fig. S4, the PVP/Ag@PU nanofiber composite membrane (10 wt% Ag concentration) had an ultralow resistance (2.07 Ω) and was rich in metallic luster. As a result, the composite organohydrogel’s conductivity with 10 wt% Ag concentration was extremely improved. When the Ag concentration was increased from 10 wt% to 15 wt%, the composite organohydrogel exhibited only 6% higher conductivity than that of 10 wt%. Simultaneously, multiple and big cracks were found on the surface of the nanofibers with 15 wt% Ag concentration, as presented in Fig. S2e, f. Considering that the excessive Ag concentration causes the agglomeration of AgNPs on the nanofibers, the AgNPs cannot be uniformly distributed on the membrane with 15 wt% Ag concentration, which can disrupt the continuity of the conductive layer and be disadvantageous to the stable transmission of electrical signals. Therefore, from the perspective of stability and cost saving, in this work, we choose the nanofiber with 10 wt% Ag concentration to reinforce the organohydrogel.

To further elaborate the sandwich-like structure of the NCRO, the cross-sectional image of the composite organohydrogel could be observed in Fig. 1f. It was evident that the top and bottom gel layers exhibited the same thickness of ~ 100 μm which is slightly lower than that of the nanofiber composites (~ 120 μm). As shown in Fig. 1e, unlike the original nanofiber composite membrane, the Ag nanoparticles, which should be well-defined and uniformly distributed on the nanofibers, showed blurred edges after being coated with the organohydrogel, and many fibers are physically bonded tightly. Subsequently, the interfacial bonding between the organohydrogel and nanofibers was observed in Fig. 1g. The gel precursor solution penetrated inside the nanofibers through the porous structure of the nanofiber membrane to form an integrated structure. In addition, we peeled off the top gel layer to observe the interleaved nanofiber composite layer. As exhibited in Fig. S5, the AgNPs were almost fully buried by the gel and its surface became enormously smooth, quite similar to the pure PU nanofibers without any AgNPs decoration. This is due to the tight bonding of the gel layers to the nanofiber membrane layer.

### Mechanical Properties of the NCRO

**Fig. 2:**
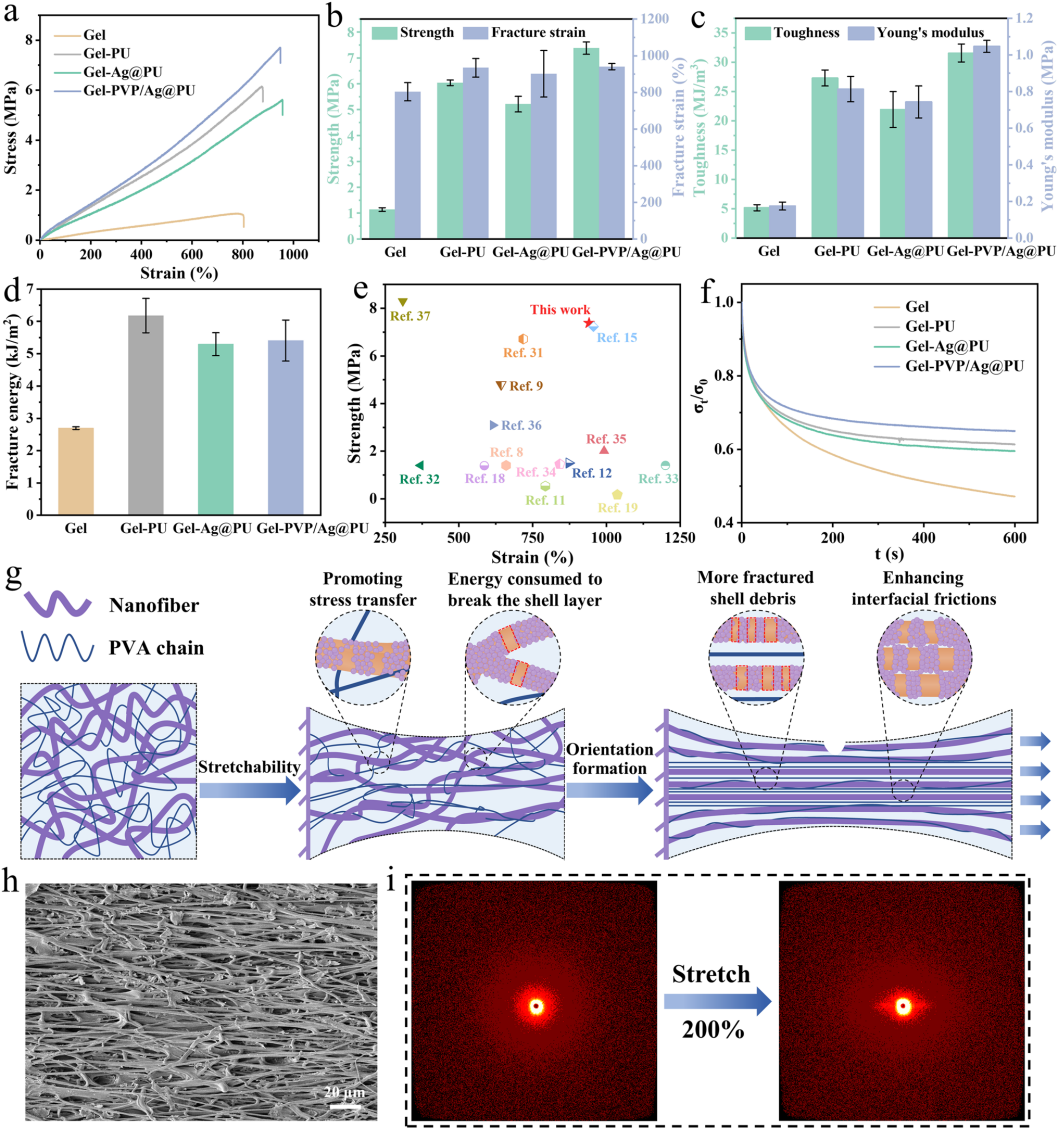
Mechanical properties of the NCRO. **a** Tensile stress-strain curves of Gel, Gel-PU, Gel-Ag@PU and Gel-PVP/Ag@PU. **b** Strength and fracture strain and **c** toughness and Young’s modulus of different gels. **d** Fracture energy of various organohydrogels. **e** Comparison of our composite organohydrogels with other organohydrogels by strength versus strain. **f** Stress-relaxation curves for different gel samples. **g** Schematic illustration of the strengthening and toughening mechanism. **h** SEM image of the nanofiber composite membrane interlayer of the NCRO stretched by 200% strain. **i** SAXS patterns of the Gel during in-situ stretching

To facilitate descriptions and discussions, the PVA/glycerol/water organohydrogel is designated as Gel. As shown in Fig. 2a, the typical stress-strain curves of Gel and composite organohydrogels consisting of different nanofiber membrane interlayers showed that the mechanical properties of organohydrogels were greatly improved after Gel was compounded with the nanofiber membranes. The detailed mechanical properties of strength, fracture strain, toughness and Young’s modulus were summarized in Fig. 2b, c. It could be observed that the tensile strength and fracture strain of Gel were only 1.14 ± 0.07 MPa and 804 ± 50%, respectively. Nevertheless, Gel-PVP/Ag@PU possessed a tensile strength of 7.38 ± 0.24 MPa and fracture strain of 941 ± 17%, which were much higher than those of Gel. This can be attributed to the dense network structure constructed by multiple non-covalent interactions in Gel-PVP/Ag@PU. As exhibited in Fig. 2c, the toughness of Gel-PVP/Ag@PU reached up to 31.59 ± 1.53 MJ m^−3^, and yet Gel merely possessed a toughness of 5.19 ± 0.53 MJ m^−3^. Moreover, Gel-PVP/Ag@PU had the Young’s modulus of 1.05 ± 0.03 MPa, 6 times higher than that of Gel. Subsequently, the dynamic viscoelasticity tests of Gel and composite organohydrogels were performed. For the organohydrogels, the storage modulus (*G'*) was always higher than the loss modulus (*G"*) in the linear region, proving the solid-like and elastic nature of the organohydrogels (Fig. S6a). Moreover, Gel-PVP/Ag@PU exhibited higher *G'* and *G"* than other organohydrogels in the strain range from 0.01% to 100% (Fig. S6b), indicating a strong intermolecular interactions in the gel. Furthermore, Gel-PVP/Ag@PU could also withstand the behaviors of twisting, rolling and folding, proving its outstanding softness and flexibility in addition to high strength (Fig. S7).

For Gel-PU, the tensile strength was 6.04 ± 0.11 MPa with the fracture strain of 934 ± 51%, higher than that of Gel-Ag@PU (5.21 ± 0.30 MPa) with the fracture strain of 902 ± 127% (Fig. 2b). After the rigid AgNPs were introduced, the mechanical properties of Gel-Ag@PU did not improve compared to Gel-PU, but decreased. This indicates that the strength at the interfaces between AgNPs and PU nanofibers as well as between AgNPs and the organohydrogel is relatively weak, resulting in Gel-Ag@PU not being able to transfer the stress to AgNPs effectively and the stress not being uniformly distributed when Gel-Ag@PU is subjected to tensile deformation. Whereas, the addition of PVP imparted Gel-PVP/Ag@PU an improved strength. The introduction of PVP strengthens the interfacial bonding between AgNPs and PU nanofibers, and the hydrogen bonds between PVA and PVP may be formed at the same time, thus achieving efficient stress transfer during the stretching process. Due to the core-shell structure between PVP and AgNPs, the motion of macromolecules is not confined by the rigid AgNPs, which guarantees the stretchability of Gel-PVP/Ag@PU. In practical applications, we usually use the fracture energy to assess the resistance of a material to cracking. It could be found from Figs. 2d and S8-S9 that the fracture energy for Gel-PVP/Ag@PU was determined to be 5.41 ± 0.63 kJ m^−2^, which was higher than that 2.70 ± 0.04 kJ m^−2^ for Gel while slightly lower than that (6.18 ± 0.53 kJ m^−2^) for Gel-PU. The high resistance of Gel-PVP/Ag@PU to tearing is attributed to large force transfer lengths generated by the embedded nanofiber composite membrane through high fiber/matrix modulus ratios, thus preventing further crack propagation [29, 30]. As shown in Video S1, Gel-PVP/Ag@PU did not delaminate in the pure shear test, suggesting that the excellent interfacial bonding of the gel to the membrane.

We further compared the tensile strength and fracture strain of our composite organohydrogel with other organohydrogels from literatures [8, 9, 11, 12, 15, 18, 19, 31–37]. For organohydrogels, two significant parameters, strength and fracture strain, are frequently used for comparison of the mechanical properties. As exhibited in Fig. 2e, the composite organohydrogel in this work shows outstanding tensile strength, higher than most organohydrogels. Although the fracture strain of few organohydrogels, such as PVA/PVP/glycerol/CaCl_2_ and PVA/CNF/TA/glycerol/NaCl, is slightly higher than that of our composite organohydrogel, they are both far less strong than our gel. Moreover, the comparative details of tensile strength, fracture strain and toughness of organohydrogels are given in Table S1. It can be noted that the composite organohydrogel also shows the excellent toughness among different kinds of organohydrogels listed in the table. In a word, the NCRO in this work exhibits superior mechanical properties to most organohydrogels.

Stress relaxation tests were carried out to demonstrate the high elasticity of the NCRO (Fig. 2f). When all samples were stretched to 100% strain, Gel showed the maximum stress relaxation over a holding time of 600 s. This indicates that the existence of the PU elastic nanofiber network can ensure the resilience of the organohydrogels to the maximum extent after being stretched by external forces. As shown in Fig. S10, the residual stresses of Gel, Gel-PU, Gel-Ag@PU and Gel-PVP/Ag@PU were 47%, 61%, 60% and 65%, respectively. Interestingly, Gel-PVP/Ag@PU exhibited a higher stress retention than Gel-PU and Gel-Ag@PU. This is attributed to the multi-layer network structure constructed by multiple non-covalent interactions and the existence of the excellent interfacial bonding in the sandwich-like structure. Therefore, due to the strengthening and toughening effect of the elastic nanofiber network and the effective stress transfer of the multi-layer network structure, Gel-PVP/Ag@PU exhibited highly elastic properties.

The excellent mechanical properties of Gel-PVP/Ag@PU including high strength and toughness may originate from the strengthening and toughening effect of the nanofiber composite in the NCRO. As shown in Fig. 2g, owing to the synergistic effect of the freezing induced macromolecular chain aggregation, interactions for polymer-solvent-polymer, and interactions of PVA chains with the multi-layer core-shell structure, the mechanically interlocked network structure of the composite organohydrogel is formed [38]. When the composite organohydrogel is stretched and deformed, both the curly PVA chains and nanofibers, which are intertwined with each other, change from a random to aligned state in the same direction. The morphology of the nanofiber composite membrane interlayer in the sandwich-like structure after stretching 200% strain was observed in Fig. 2h, and the stretch-induced fiber orientation was observed. Compared to the unstretched membrane in Fig. S11, after being stretched, the membrane showed a denser and smaller porous structure. This is conducive to reducing stress concentration and hence preventing the crack propagation and improving the mechanical properties [39]. Moreover, the rough interface between the AgNPs decorated PU nanofibers and PVA chains promotes stress transfer [40]. When the PVA chains are deformed, they can transfer the stress to the rigid AgNPs and elastic PU nanofibers through strong interfacial bonding and thus the NCRO can become stronger and tougher. Furthermore, with the stretching of the NCRO, a vast amount of energy is consumed by the fracture of the PVP-Ag shell layer on the nanofiber composite, contributing to the improvement of the mechanical properties. The separated PVP-Ag shell layer formed by fracture on one nanofiber contacts with the bare PU core layer on another nanofiber, forming a gear-like structure. Thus, the exposed AgNPs enhance interfacial frictions between the aligned nanofibers. At the same time, the numerous newly formed fractured PVP-Ag shell layer requires the continued rupture of the previous broken shell layer, a process that consumes more energy. This indicates that further stretching of the NCRO enhances the mechanically interlocked structure, effectively improving the mechanical properties. In addition, as indicated by the SAXS results in Fig. 2i, obvious macromolecular alignment was exhibited from 0 to 200% strain, demonstrating the stretching induced macromolecular alignment in the gel layers. Hence, the nanofibers in the membrane layer and the nanofibrils in the gel layers can enhance the fracture toughness of the composite organohydrogel by combination of the fiber pulling out and bridging mechanism [41]. Simultaneously, enormous amounts of energy are dissipated to break the non-covalent interactions such as hydrogen bonds of PVA to PVP, and PVA to PU during the fracture process, hence guaranteeing high strength and toughness of the composite organohydrogel. In brief, due to PU nanofiber composite orientation and strong interfacial interaction (micro-scale), PVA nanofibril orientation (nano-scale) and non-covalent interactions (molecular-scale), the synergistic strengthening and toughening mechanism at three different length scales is responsible for the excellent mechanical properties of the composite organohydrogel.

To investigate the energy dissipating mechanism, the cyclic tensile tests of the organohydrogels were further performed. As shown in Fig. S12, the Mullins effect, as a typical feature of soft matters, could be observed in the dependence of the organohydrogels’ stress on the loading history. All the three organohydrogels were subjected to continuous loading-unloading experiments in the strain range of 0–500% with a step increase of strain (100%). A clear hysteresis loop was observed under the stress-strain curve associated with each 100% strain increment, and the hysteresis loop became larger as the strain gradually increased. This suggested that the physical interactions in the organohydrogels could effectively dissipate the energy with increasing tensile strain. In addition, the fatigue resistance of Gel, Gel-PU and Gel-PVP/Ag@PU was studied through 1000 continuous stretching-releasing cycles with a fixed strain (100%). Obviously, Gel-PVP/Ag@PU exhibited the most evident hysteresis loop than Gel and Gel-PU in the first cycle, which was attributed to the rapid dissociation of physical interactions in the composite organohydrogel network and the formation of the oriented structure of PU nanofibers and PVA nanofibrils during stretching induced deformation (Fig. S13). As exhibited in Fig. S14, after 50 cyclic tensile tests without resting time between each cycle, the maximum stress of Gel, Gel-PU and Gel-PVP/Ag@PU dropped by 25%, 15% and 14%, respectively. In the 50th to 1000th cycles, the trend of stress variation became smaller for all organohydrogels. It could be noted that Gel-PVP/Ag@PU retained a tensile stress of 0.49 MPa after 1000 cyclic tests at 100% strain, higher than that 0.35 MPa and 0.09 MPa of Gel-PU and Gel, respectively. The result demonstrates the existence of strong physical interactions in the composite organohydrogel network. Furthermore, the corresponding dissipated energy of Gel-PVP/Ag@PU remained essentially unchanged during the 50^th^ to 1000^th^ cycles, proving the composite organohydrogel’s excellent fatigue resistance.

### Electrical Properties of the NCRO

In practical applications, it is extremely significant for materials to maintain durability over a wide temperature range and under mechanical deformation. As shown in Fig. S15a, the normalized relative conductivity variation (*σ*/*σ*_0_) of the NCRO was measured as a function of the room-temperature storage days, where *σ*_0_ and *σ* refer to the initial and durability-tested conductivity, respectively. The NCRO decreased slightly in the conductivity after being placed for one day at room temperature, and the retention of its conductivity exhibited a fluctuation around 80% for the next six days. Also, it could be seen from the inset that the NCRO presented no obvious change in color. The stable conductivity could be attributed to the protective effect of the PVP layer in the core-shell structure and the gel layers in the sandwich-like structure, greatly decreasing the possibility of the AgNPs being oxidized. Subsequently, we conducted the durability tests of the NCRO at extreme temperatures, as presented in Fig. S15b. It could be found that after 7 days at a low temperature of − 25 °C and 2 days at a high temperature of 60 °C, the NCRO as a whole could maintain more than 82% and 70% of the original conductivity, respectively. Due to the existence of the glycerol, the growth of ice crystals inside the NCRO was effectively inhibited at a low temperature, endowing it with excellent anti-freezing properties. In addition, the result of the rheological test revealed that the *G'* and *G"* remained essentially unchanged in the range of 25 to 60 °C, demonstrating the high temperature resistance of the NCRO (Fig. S16). Furthermore, the *σ*/*σ*_0_ of the NCRO under different stretching-releasing cycles (20% strain) was investigated in Fig. S15c. It could be observed that the conductivity dropped by about 30% after the first 100 cycles, yet the *σ*/*σ*_0_ fluctuated around the value of 0.65 in the next 900 cycles. This indicated that the NCRO could quickly recover its continuous conductive network after being stretched.

**Fig. 3:**
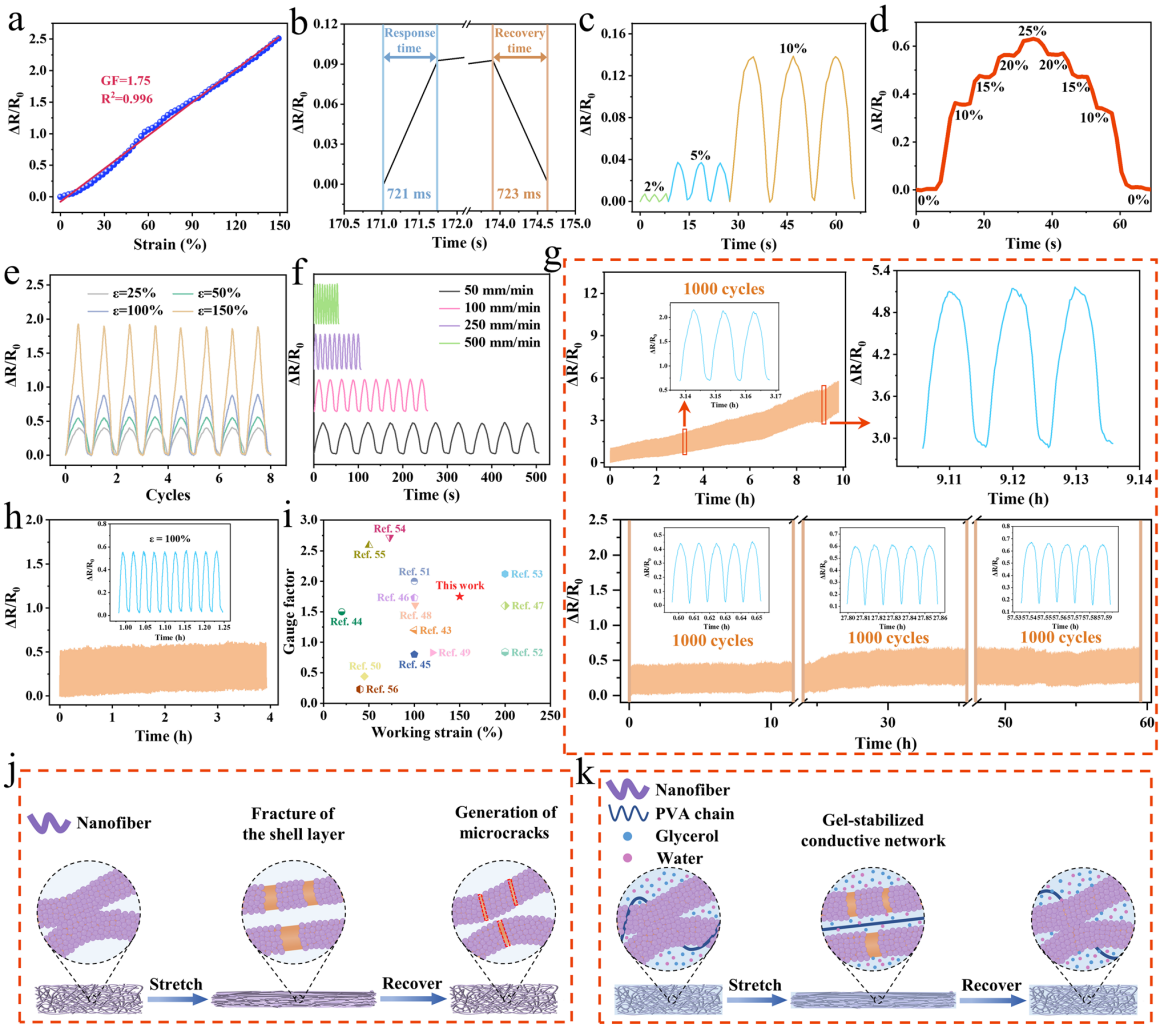
Strain sensing properties of the NCRO. **a** Relative resistance-strain response curves. **b** Response time and recovery time of the NCRO at 10% strain. **c** Relative resistance changes for small strains (2–10%). **d** Relative resistance variations of the sensor gradually stretched to 10%, 15%, 20% and 25%. **e** Relative resistance changes for large strains (25–150%). **f** Relative resistance response at different tensile rates under the strain of 100%. **g** Comparison between the 1000 cyclic strain sensing performance of the nanofiber composite membrane and the 3000 cyclic strain sensing performance of the NCRO at 30% strain. The insets are several random cyclic sensing curves. **h** The 150 cyclic strain sensing performance of the sensor at 100% strain. Inset is the random 10 cyclic sensing curves. **i** Comparison of the sensor in this work to literatures by gauge factor versus working strain. **j** Schematic diagram of the pure nanofiber composite membrane sensing mechanism. **k** Schematic illustration of the mechanism for the gel-stabilized conductive network

The excellent mechanical properties and high conductivity make it possible to use the NCRO as a wearable strain sensor. To evaluate the strain sensitivity of the NCRO strain sensor, the gauge factor (GF) is calculated from the slope of the fit line, which is derived from the plot of the relative resistance change (Δ*R*/*R*_0_) versus strain (*ε*). As presented in Fig. 3a, the relative resistance change increased monotonously with the increase of the strain and only exhibited a linear responsive region, i.e., a value of 1.75 for GF in the whole strain range of 0%-150%, which is quite desirable and important in practical applications, because it can provide accurate, reliable and consistent sensing signals regardless of the strain. Moreover, the sensitivity of the composite organohydrogel was further investigated in Fig. 3b. It could be found that the NCRO possessed a response time of 721 ms and a recovery time of 723 ms under 10% strain at a stretching rate of 500 mm min^−1^. Figure 3c showed that the sensor could monitor small strains from 2 to 10%, where different applied strains corresponded to different response signals. As shown in Fig. 3d, we explored the electrical stability in the stair-type strain range of 0–25%. The relative resistance maintained the same stepwise increasing trend as the strain and its value did not change during the 5-second retention time of each shift. Furthermore, the dynamic tensile tests of large strains (25%-150%) for the NCRO were carried out (Fig. 3e). The stable and repeatable resistance response curves confirmed the reliable working capability of the sensor. These results revealed that the sensing signals output by the NCRO strain sensor was recognizable, accurate and stable. Subsequently, the effect of different motion frequency on the relative resistance response was observed at different tensile rates (50–500 mm min^−1^) under the strain of 100% (Fig. 3f). It could be noted that little variation occurred between the sensing signals, and thus the strain sensing behavior was independent of the tensile rate, facilitating accurate and stable monitoring of human movements.

For a flexible wearable strain sensor, reproducibility and stability are essential during the long-term stretching and releasing process. All cyclic strain sensing tests in this work were performed after the samples were pre-stretched for 150 cycles at a fixed strain. As shown in Fig. 3g, the 1000 cyclic strain sensing performance of the nanofiber composite membrane at 30% strain was first studied. It was clear that the sensing signals of the nanofiber composite appeared to be extremely unstable with the continuous increase of the Δ*R*/*R*_0_ during the cycles. Then, the cyclic durability test of the NCRO strain sensor for 3000 cycles was performed under 30% strain at a tensile rate of 20 mm min^−1^. Apparently, the relative resistance changes did not show significant fluctuation throughout the cyclic stretching-releasing process, which could be observed from the insets. The enormously unstable sensing signals of the membrane in 1000 cycles and the excellent sensing stability of the NCRO in 3000 cycles are evidence that the NCRO has a greater advantage over the membrane when applied as the sensor in long-term use scenarios. The excellent sensing stability of the NCRO can be attributed to the filling effect of the gel on the nanofiber network during the stretching-recovering process (Fig. S17). Moreover, we performed the weight loss experiment with the NCRO left in the environment for 60 h (Fig. S18). Due to the hygroscopic nature of glycerol, its low vapor pressure and its ability to form hydrogen bonds with water molecules [42], the NCRO started to absorb water vapor from the air to increase its weight during the first hour. After that, the weight loss of the NCRO increased and remained stable after 12 h. Hence, due to good elasticity of PU nanofibers and the gel-stabilized conductive network, the gel based sensor possesses outstanding cyclic and long-term sensing stability and durability. Not only at a small strain, but also at a large strain of 100%, the sensor still exhibited excellent sensing response stability during 150 cycles (Fig. 3h). As shown in Fig. 3i, we compared the sensing performance of our composite organohydrogel based sensor with other gel based sensors by the GF versus working strain [43–56]. The detailed comparison of the GF, working strain and sensing stability were summarized in Table S2. It can be found that the NCRO exhibits good sensitivity over a wide strain range and outstanding long-term sensing stability.

We investigated the sensing mechanisms of the nanofiber composite membrane and NCRO, which are schematically demonstrated in Fig. 3j, k. During the stretching of the nanofiber composite membrane, the AgNPs distributed on the PU nanofibers change from a complete and continuous state to a broken shell layer (PVP-Ag layer), making the electrical signal transmission more difficult and hence increasing the resistance. Moreover, the nanofiber composite exhibits a significant increase in length and decrease in cross-sectional area, which likewise has an impact on the resistance of the nanofiber composite strain sensor. For the NCRO, the presence of the gel matrix can limit the fracture of the shell layer. As shown in Fig. S5, the pores inside the nanofiber composite were filled with the gel that can protect the conductive network from deformation to some extent. Thus, the response intensity of the NCRO is relatively small compared to that of the nanofiber composite membrane. During the recovering of the nanofiber composite membrane, it is difficult for two nanofibers that have been pulled apart by external forces to return to the initial state of mutual contact, and it may be accompanied by the generation of microcracks on the surface of the nanofibers. This is very detrimental to the long-term sensing stability of the nanofiber composite membrane. Fortunately, the existence of the gel provides the impetus for the nanofibers to revert, and the gel does its best to keep the nanofibers in their initial state, which guarantees the stability of the NCRO strain sensor in the long-term use. In addition, owing to the nature of the mechanically interlocked structure of the NCRO, the PVA, glycerol and water in the gel layers penetrate the interior of the nanofiber composite membrane through the pores in the membrane, thus forming a protective barrier of the membrane against the air. The isolation of the membrane from air greatly reduces the chance of silver oxidation, which is also advantageous for the formation of a stabilized conductive network. Therefore, compared with the nanofiber composite membrane, the NCRO enables more excellent long-term sensing stability and durability.

**Fig. 4:**
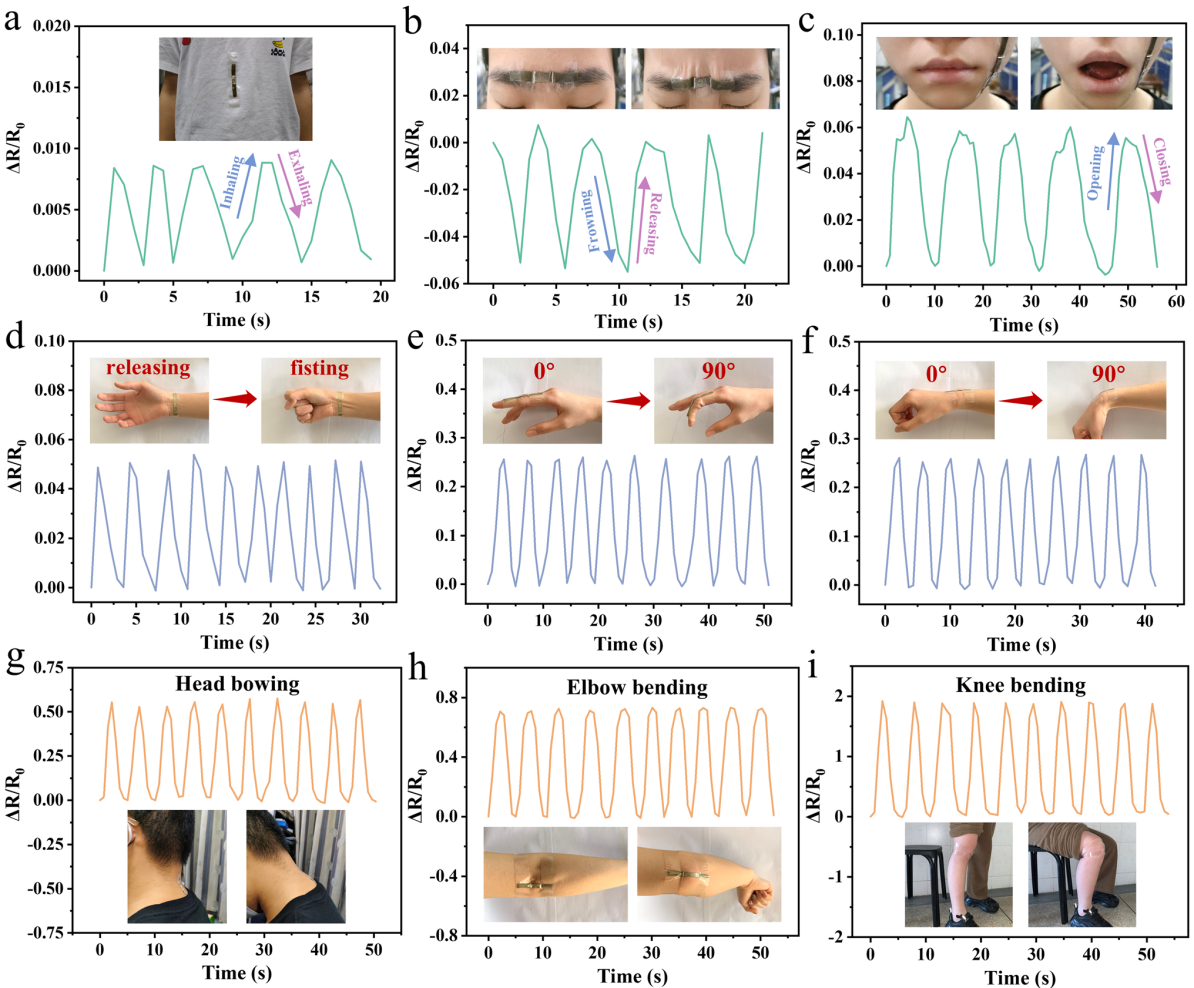
Human motion detections of the NCRO strain sensor. **a** Breathing. **b** Frowning. **c** Opening the mouth. **d** Making a fist. **e** Finger bending. **f** Wrist bending. **g** Head bowing. **h** Elbow bending. **i** Knee bending

By virtue of the remarkable sensing performance, the NCRO exhibits a great application prospect as a wearable strain sensor to detect human motions. To obtain instant response signals, the NCRO strain sensor was attached on different parts of a volunteer’s body. As shown in Fig. 4a, the sensor was applied to monitor subtle human motions, such as respiration, in a way of installing the sensor on the tummy. It could be observed that the relative resistance changes increased or decreased correspondingly with each inhalation or exhalation of the volunteer, respectively. Also, the facial expressions of frowning and opening the mouth could be accurately detected by the NCRO strain sensor (Fig. 4b, c). Different expressions corresponded to different response intensities, and the sensor had repeatable sensing response, which indicated the reliable working ability of the sensor. Moreover, sensing signals for clenching and releasing could be readily captured by the sensor, as presented in Fig. 4d. This laid the foundation for the design of wearable sensors that could monitor the recovery of injured wrists. As exhibited in Fig. 4e, f, when the finger and wrist were bent at 90°, the corresponding relative resistance variations peaked simultaneously. Then, the relative resistance values promptly returned to its original levels when the finger and wrist kept straight. In addition, the NCRO strain sensor possessed the capability of monitoring large movements, such as head bowing, elbow bending and knee bending (Fig. 4g, h, i). The relative resistance changes between different motions varied immensely, implying the discriminative nature of the sensor. Based on these results, the NCRO could be a promising flexible wearable strain sensor for practical use.

**Fig. 5:**
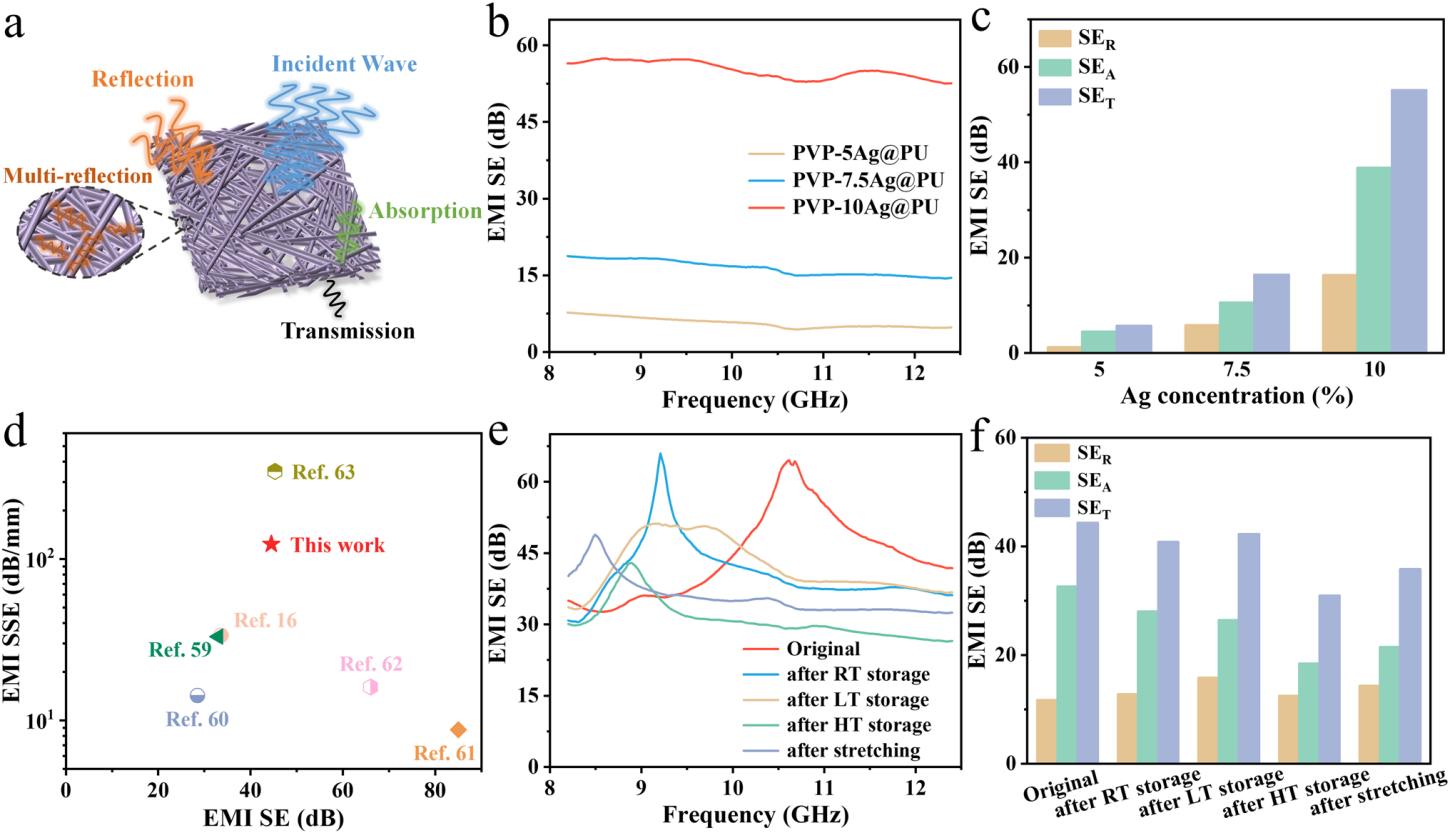
EMI shielding properties of the NCRO. **a** Schematic illustration of the EMI shielding mechanism. **b** EMI SE of the nanofiber composite membranes with different Ag concentrations in the X band. **c** The average SE_T,_ SE_A_ and SE_R_ of PVP-XAg@PU. **d** Comparison of the composite organohydrogel in this work to other organohydrogels and hydrogels by EMI specific shielding effectiveness (SSE) versus EMI shielding effectiveness (SE). **e** EMI SE of the original composite organohydrogel and the composite organohydrogel after different durability tests. **f** The average SE_T_, SE_A_ and SE_R_ of the composite organohydrogel before and after different durability tests

The high conductivity imparted by AgNPs also allows the NCRO to exhibit the excellent EMI shielding properties. Since the electrically conductive nanofiber composite plays a dominant role in determining the EMI shielding performance of the NCRO with the sandwich-like structure, we first explored the shielding performance of the nanofiber composite membrane. The EMI shielding performance is mainly determined by the reflection, absorption and multiple reflections, which is schematically illustrated in Fig. 5a. We can qualitatively evaluate the capabilities of the EMI reflection, absorption and multiple reflections by the mobile charge carriers, electric dipoles and internal structure, respectively [57]. As known, the reflection occurs mainly on the surface of the material, so when the material’s surface carries a large number of free electrons, it facilitates a large reflection of the incident electromagnetic wave [58]. That is to say, the high conductivity of the membrane may favor the incident wave to be reflected in large quantities. Moreover, the large conductivity mismatch caused by the conductive Ag layer and the insulating PVP layer and PU layer in the core-shell structure is conducive to the charge polarization, which promotes the absorption of the electromagnetic wave entering the membrane. Simultaneously, the porous structure of the nanofiber composite membrane allows more incoming electromagnetic waves to be trapped, thus enabling multiple reflections. As shown in Fig. 5b, c, as the Ag concentration increased, the values of SE_R_, SE_A_ and SE_T_ increased accordingly, where SE_R_ and SE_A_ refer to the shielding effectiveness of the reflectivity and absorptivity, and SE_T_ refers to the total EMI shielding effectiveness. Obviously, PVP-10Ag@PU showed the highest EMI SE of 55.2 dB. As a result, the nanofiber composite membrane, as a functional interleaf for reinforcing the organohydrogel, exhibits outstanding EMI shielding performance.

Relying on the outstanding EMI shielding properties of the nanofiber composite membrane, our composite organohydrogel shows a high EMI SE of 44.5 dB with a sample thickness of 360 μm for testing (Fig. 5e, f), which is slightly lower than that of the nanofiber composite. As the organohydrogel is compounded with the membrane, the gel precursor solution penetrates into the interior of the membrane through the pores in the membrane, which, to a certain extent, affects the value of the conductivity, and thus the value of the SE. Moreover, by calculation, the specific shielding effectiveness (SSE = SE/thickness) of the composite organohydrogel is 123.6 dB mm^−1^. Hence, we compared the composite organohydrogel in this work to other organohydrogels and hydrogels by EMI SSE versus EMI SE (Fig. 5d) [16, 59–63], and the detailed information was shown in Table S3. It could be seen that our composite organohydrogel exhibited superior EMI shielding properties. In addition, it is necessary to maintain good durability when the composite organohydrogel is used in real-life applications as a wearable electronic. Figure 5e showed the EMI SE of the composite organohydrogel before and after different durability tests. The detailed SE_R_, SE_A_ and SE_T_ were summarized in Fig. 5f. Apparently, the EMI SE after low-temperature and room-temperature storage for 7 days was 42.3 and 40.8 dB, respectively. Compared to the EMI SE value of the original sample, the slight decrease signified the excellent anti-freezing performance and environmental stability. Although the SE showed a decreasing trend after different durability tests, they all remained above 30 dB at the same time in the whole X band. All the results revealed that the NCRO possessed excellent environmental tolerance, exhibiting the great potential applications in multifunctional and wearable electronics.

## Conclusions

In this study, we develop a nanofiber composite reinforcement method to develop mechanically robust and multifunctional organohydrogels with strong multi-level interfacial bonding. PU nanofiber composite orientation and strong interfacial interaction at the micrometer scale, PVA nanofibril orientation at the nanometer scale and non-covalent interactions at the molecular scale are responsible for the excellent mechanical properties of the NCRO. In addition, the NCRO with outstanding environmental tolerance can be used for high-performance EMI shielding and strain sensing. In particular, the organohydrogel stabilizes the conductive network, thus guaranteeing the long-term sensing stability and durability of the NCRO. We anticipate that following this nanofiber composite reinforcement strategy, more organohydrogels could exhibit highly promising applications in flexible electronics and people’s healthcare monitoring.

### Supplementary Information

Below is the link to the electronic supplementary material.

Supplementary file1 (AVI 2341 kb)

Supplementary file2 (PDF 1903 kb)
