# Supplementary material for: Nanofiber Composite Reinforced Organohydrogels for Multifunctional and Wearable Electronics
Source: Nanomicro Lett. 2023 Jul 7;15:174. doi: 10.1007/s40820-023-01148-9 (PMC10328881; doi:10.1007/s40820-023-01148-9)
Supplement: Supplementary file 2 — Supplementary file2 (PDF 1903 kb) [file 40820_2023_1148_MOESM2_ESM.pdf]

Supporting Information for

Nanofiber Composite Reinforced Organohydrogels for Multifunctional and Wearable Electronics

Jing Wen[1, †], Yongchuan Wu[1, †], Yuxin Gao[1], Qin Su[1], Yuntao Liu[1], Haidi Wu[1], Hechuan Zhang[1], Zhanqi Liu[1], Hang Yao[1, *], Xuewu Huang[2], Longcheng Tang[3], Yongqian Shi[4], Pingan Song[5], Huaiguo Xue[1], Jiefeng Gao[1, *]

[1] School of Chemistry and Chemical Engineering, Yangzhou University, Yangzhou, 225002, P. R. China

[2] Testing Center, Yangzhou University, Yangzhou, 225002, P. R. China

[3] Key Laboratory of Organosilicon Chemistry and Material Technology of Ministry of Education, Hangzhou Normal University, Hangzhou, 311121, P. R. China

[4] College of Environment and Safety Engineering, Fuzhou University, Fuzhou, 350116, P. R. China

[5] Centre for Future Materials, University of Southern Queensland, Springfield Central, 4300, Australia

†Jing Wen and Yongchuan Wu contributed equally to this work.

*Corresponding authors. E-mail: jfgao@yzu.edu.cn (Jiefeng Gao); yaohang@yzu.edu.cn (Hang Yao)

Supplementary Figures and Tables

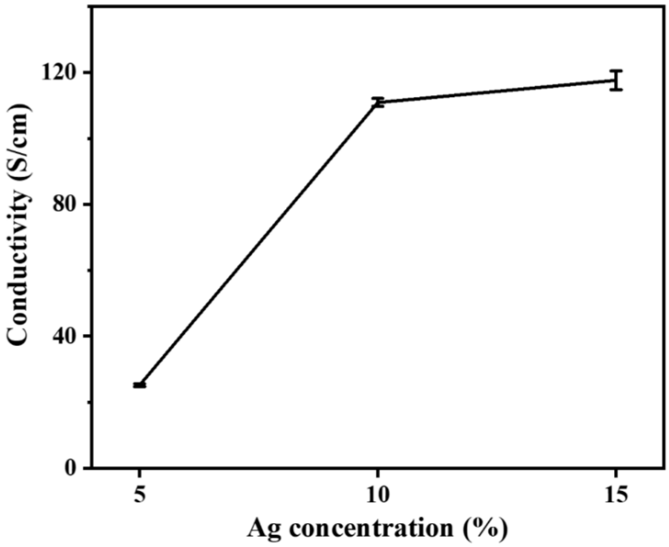

Fig. S1 Conductivity of the composite organohydrogels versus different Ag concentrations

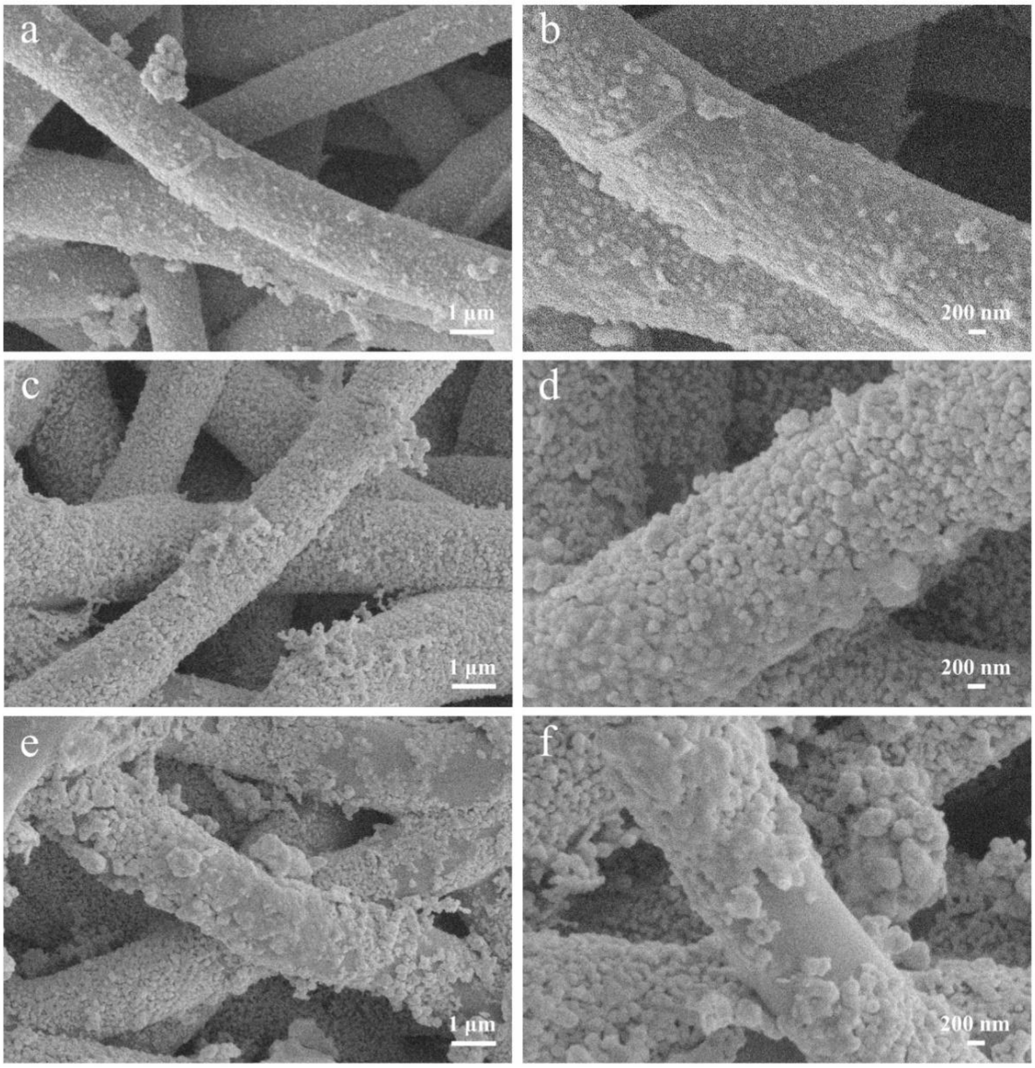

Fig. S2 SEM images of the nanofiber composite membranes with different Ag concentrations: **a** 5 wt%, **c** 10 wt% and **e** 15 wt%. **b**, **d** and **f** are the magnified SEM images of **a**, **c** and **e**, respectively

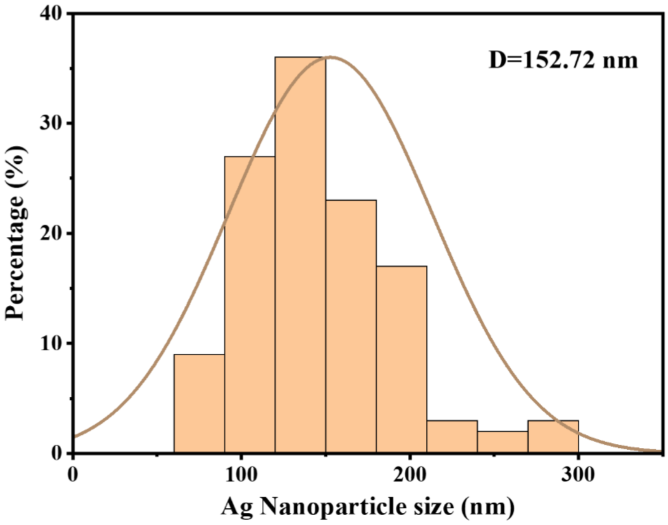

Fig. S3 Particle size distribution of AgNPs in the PVP/Ag@PU nanofiber composite membrane (10 wt% Ag concentration)

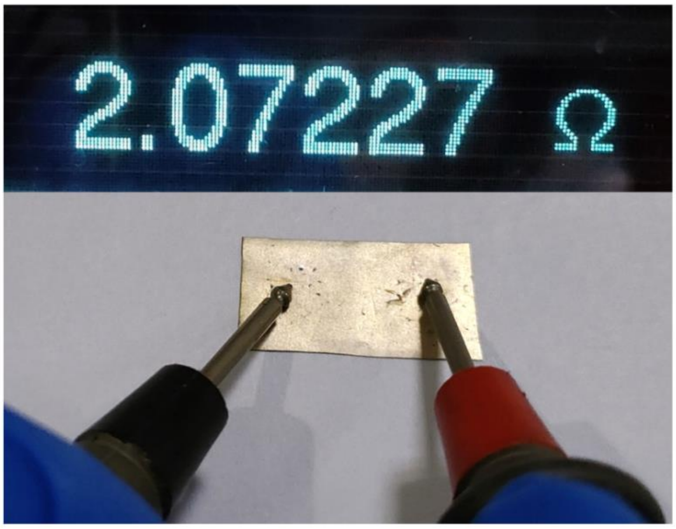

Fig. S4 The photograph of the PVP/Ag@PU nanofiber composite membrane with an ultralow resistance

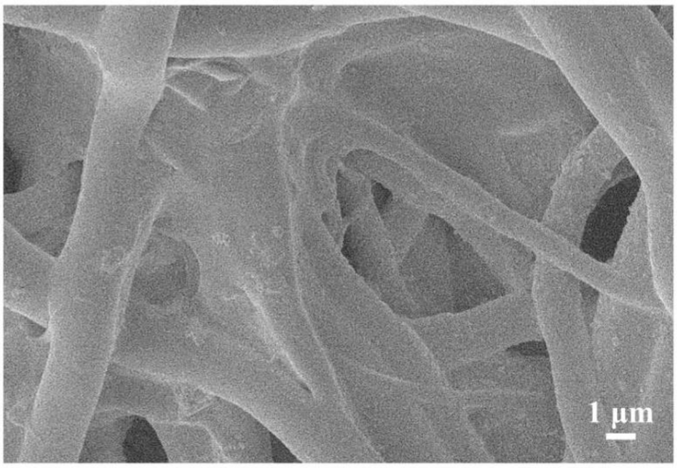

Fig. S5 Morphology of the middle nanofiber composites layer

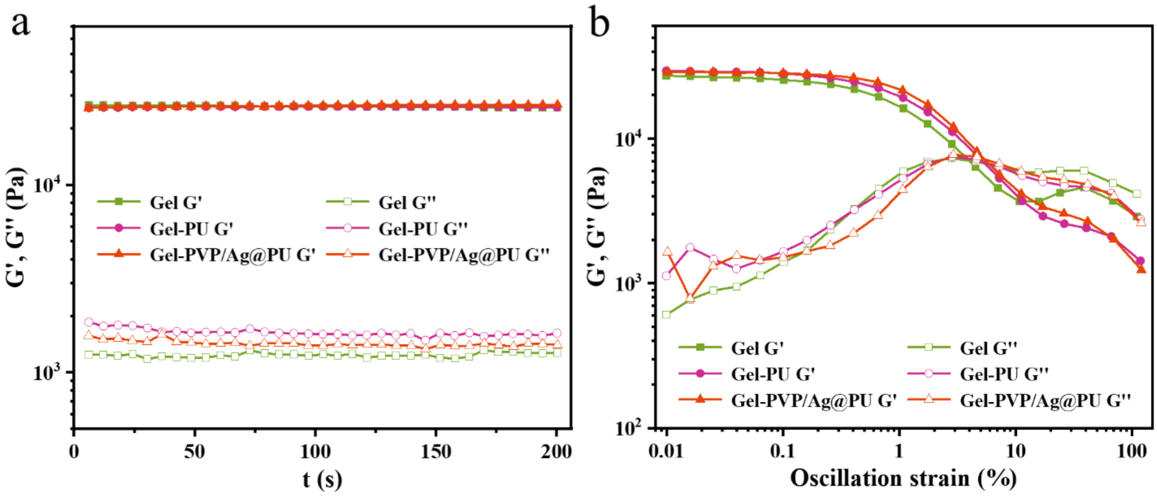

Fig. S6 The storage modulus (*G*′) and loss modulus (*G*″) of the organohydrogels as a function of **a** oscillation time (ω = 6.28 rad s^{-1}, γ = 0.1%, T = 25 °C) and **b** oscillation strain (ω = 6.28 rad s^{-1}, T = 25 °C)

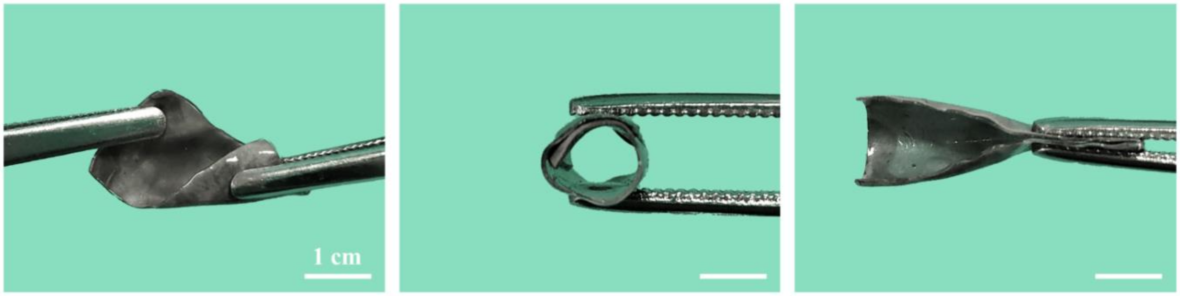

Fig. S7 Images of twisting, rolling and folding the composite organohydrogels, demonstrating the flexibility of the materials

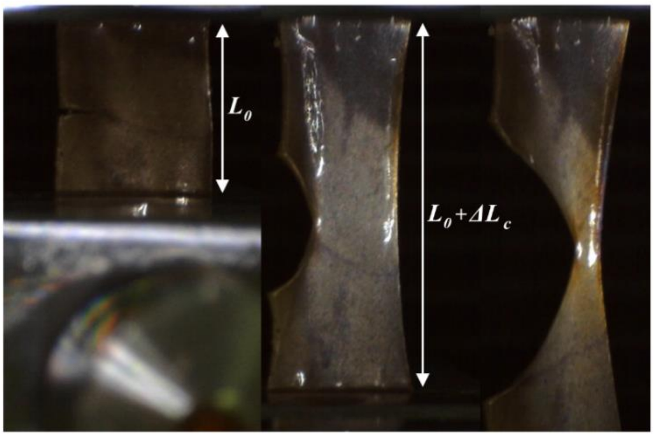

Fig. S8 The pure shear test of Gel-PVP/Ag@PU

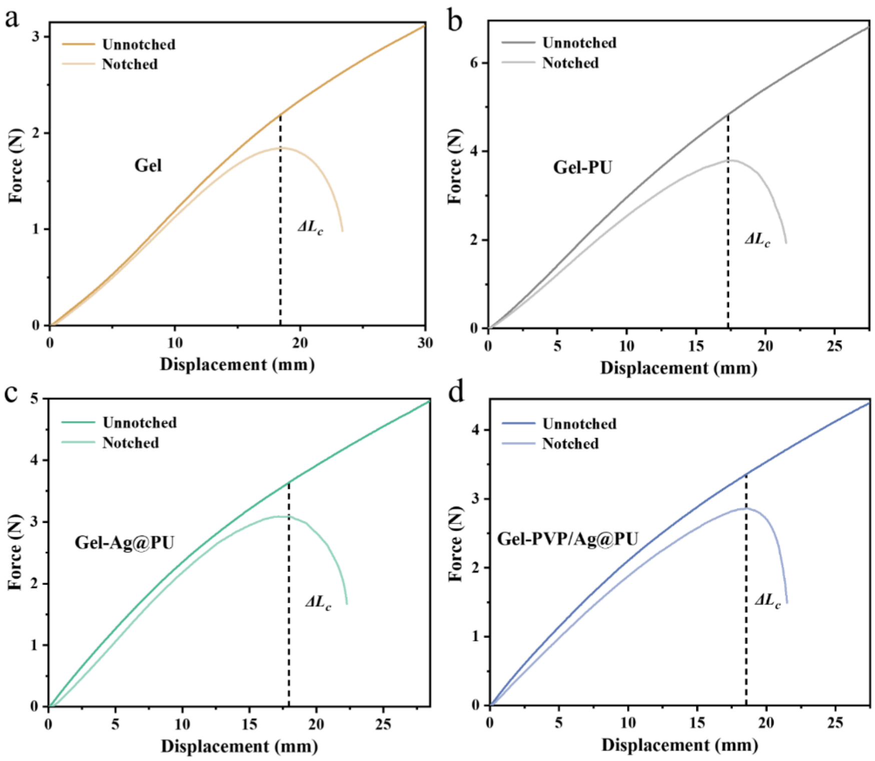

Fig. S9 Force-displacement curves of unnotched and notched **a** Gel, **b** Gel-PU, **c** Gel-Ag@PU and **d** Gel-PVP/Ag@PU

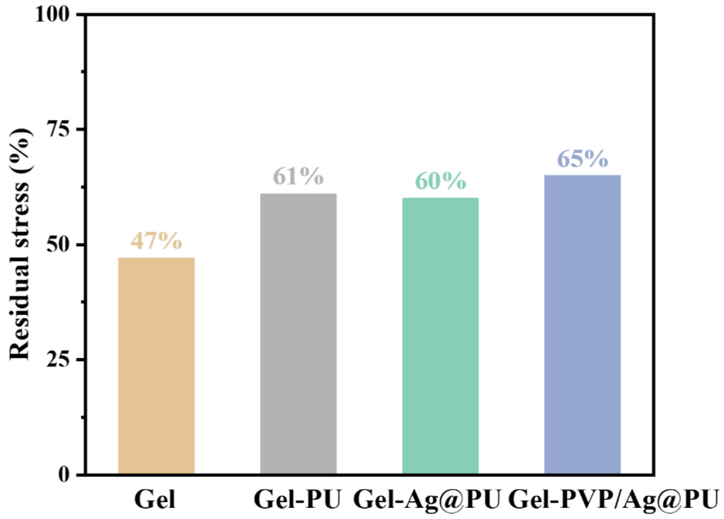

Fig. S10 Residual stresses of different gels after stress-relaxation tests

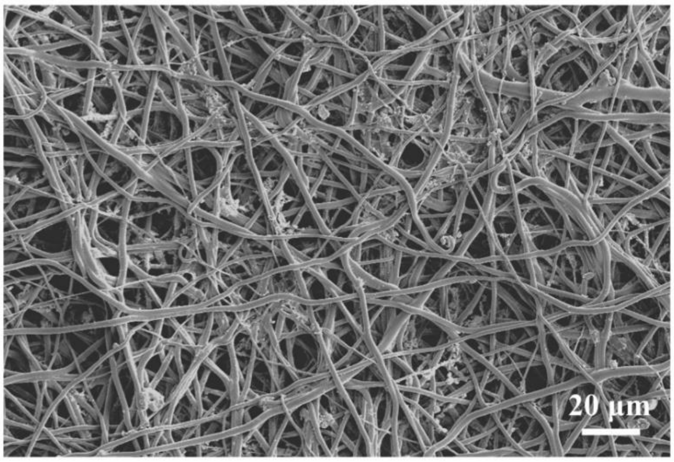

Fig. S11 The SEM image of the unstretched nanofiber composite membrane interlayer of the NCRO

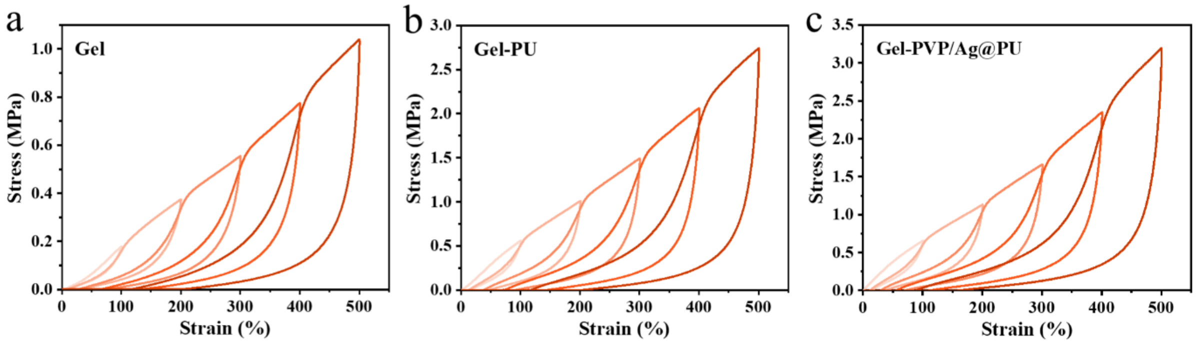

Fig. S12 Cyclic stress-strain curves of **a** Gel, **b** Gel-PU and **c** Gel-PVP/Ag@PU with 100% step increase of the strain

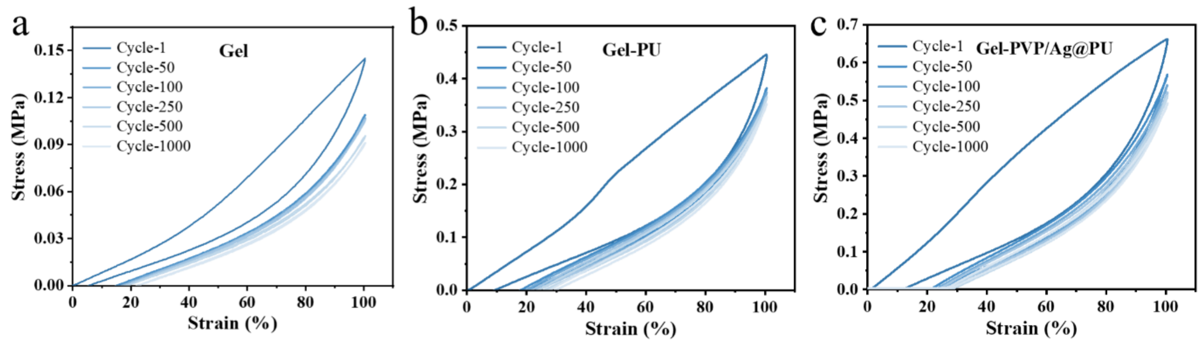

Fig. S13 Stress versus strain curves of **a** Gel, **b** Gel-PU and **c** Gel-PVP/Ag@PU with 1000 successive loading-unloading cycles (100%)

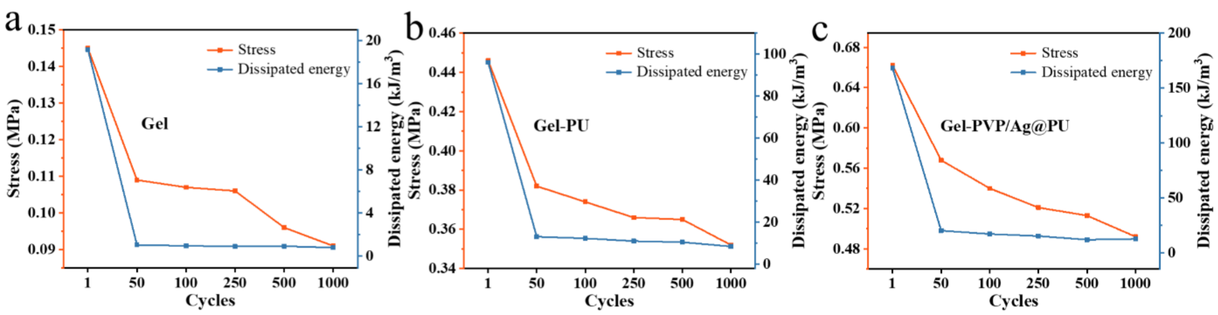

Fig. S14 The corresponding stress and dissipated energy of **a** Gel, **b** Gel-PU and **c** Gel-PVP/Ag@PU

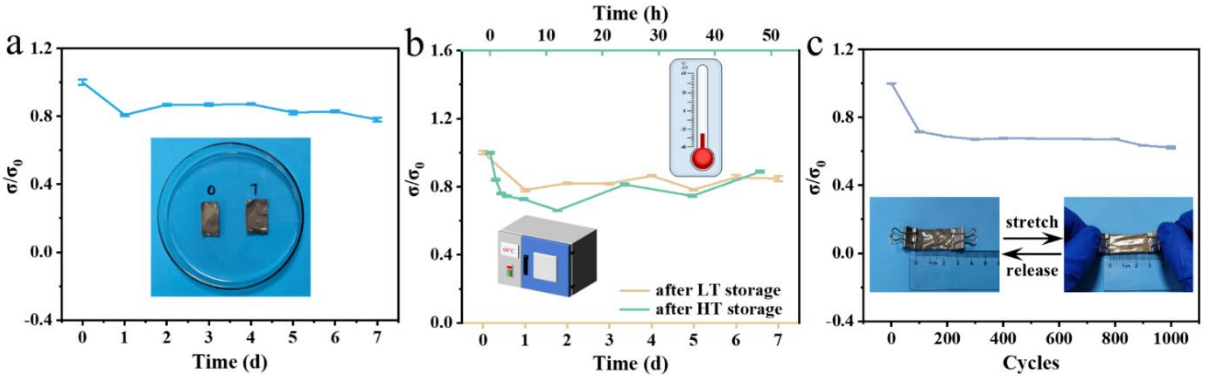

Fig. S15 Normalized relative conductivity variations of the NCRO with **a** room-temperature (RT) storage days, **b** low-temperature (LT) and high-temperature (HT) storage days, and **c** stretching cycles. The insets in each figure are the photographs demonstrating the durability tests

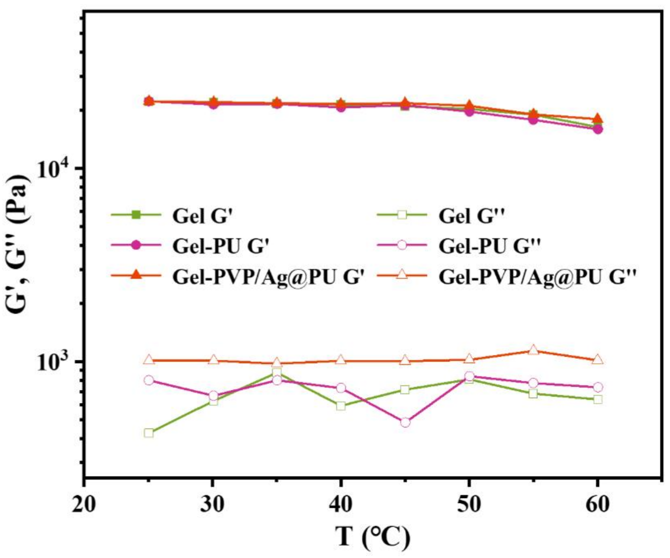

Fig. S16 The storage modulus (G') and loss modulus (G'') of the organohydrogels on a temperature sweep in the range of 25 °C to 60 °C (γ = 0.1%, ω = 6.28 rad s^{-1})

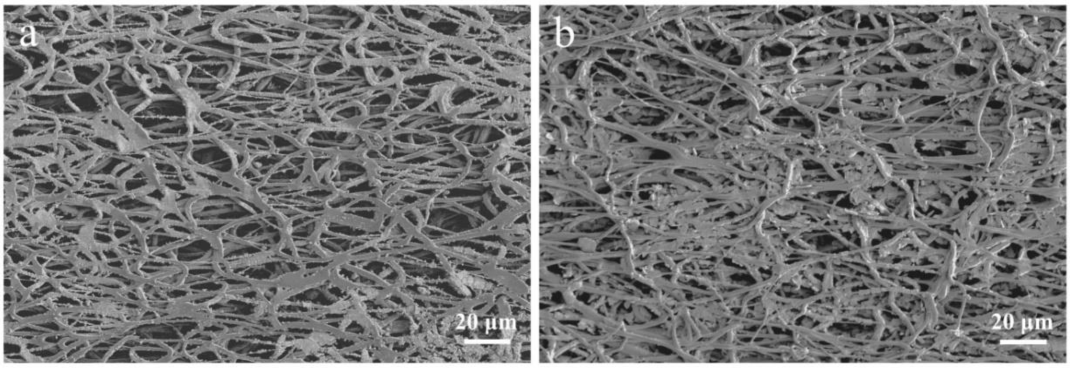

Fig. S17 SEM images of **a** the nanofiber composite membrane and **b** the nanofiber composite membrane interlayer of the NCRO (both stretched by 30% strain)

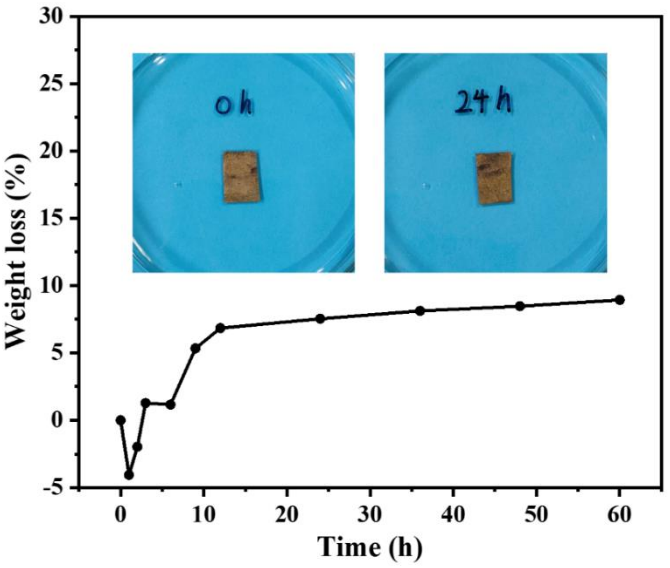

Fig. S18 Weight loss of the NCRO kept in the environment for 60 h. Inset is the photograph showing the state of the NCRO at the initial time and after 24 h

Table S1 Comparison of the composite organohydrogel in this work with other kinds of strong and tough organohydrogels

**Materials**	**Tensile strength (MPa)**	**Fracture strain (%)**	**Toughness (MJ/m^3)**	**Refs.**
PVA/glycerol/PVP/Ag@PU	7.38	941	31.59	This work
PVA/starch/glycerol	0.53	793	1.99	S1
PVA/CNF/DMSO	1.40	660	5.25	S2
PVA/glycerol/CB/CNT	4.80	643	15.93	S3
PVA/SNF/g-C_3N_4/EG	1.39	586	N/A	S4
PVA/DMSO	6.71	718	26.24	S5
PVA/glycerol/WO_3	1.50	873	6.56	S6
PVA/glycerol/NaCl	1.40	370	3.20	S7
PVA/PVP/glycerol/$CaCl_2$	1.40	1200	10.68	S8
PVA/starch/glycerol/Na_3Cit	1.45	842	6.91	S9
PVA/CNF/TA/glycerol/NaCl	2.01	992	10.41	S10
PVA/glycerol	7.23	956	36.89	S11
PAM/GE/PU/glycerol/NaCl	3.09	615	7.75	S12
PAM/PAA/MoS_2/EG	8.30	310	N/A	S13
PAM/MXene/glycerol	0.17	1037	N/A	S14

Note: “N/A” indicates “not available” in the references.

Table S2 Comparison of the sensing performance of our composite organohydrogel based sensor with other gel based sensors

Materials	GF in strain ranges	Stability (cycles)-strain	Refs.
PVA/glycerol/PVP/Ag@PU	1.75 (0-150%)	3000-30%	This work
PVA/hydroxypropyl cellulose	1.2 (0-100%)	N/A	S15
PVA/PEDOT:PSS	1.5 (0-20%)	N/A	S16
PAAM/carrageenan/glycerol	0.8 (0-100%)	N/A	S17
VSNPs/PAAm/SA	1.73 (0-100%)	2500-25%	S18
PVA/CA/AgNPs	1.6 (0-200%)	200-50%	S19
PVA/PAA/PEDOT:PSS/CNTs	0.66 (0-24%)<br>0.71 (24-58%)<br>1.61 (58-101%)	N/A	S20
PVA/PAANa	0.83 (0-120%)	N/A	S21
PAAM/carrageenan/LiBr	0.44 (0-45%)	700-45%	S22
PVA/NaCl	1.35 (0-1%)<br>1.7 (1-10%)<br>2.0 (10-100%)	200-30%	S23
PAAm/PAAc/PDA/NaCl	0.44 (0-60%)<br>0.69 (60-140%)<br>0.84 (140-200%)	N/A	S24
DMAEA-Q/NaSS/CNFs/CNTs	1.02 (0-60%)<br>1.4 (60-140%)<br>2.12 (140-200%)	2000-100%	S25
PAA/sodium lignosulfonate/SA	2.72 (0-72.8%)	200-20%	S26
PVA/TA/EGaIn/NaCl	2.59 (0-50%)	800-20%	S27
PAA/TA/CNC	0.23 (0-40%)	1000-55%	S28

Note: “N/A” indicates “not available” in the references.

Table S3 Comparison of the EMI shielding performance between the composite organohydrogel in this work and other organohydrogels and hydrogels reported in literatures

Materials	EMI SE (dB)	Thickness (mm)	EMI SSE (dB/mm)	Refs.
PVA/glycerol/PVP/Ag@PU	44.5	0.36	123.6	This work
PVA/PAAm/MXene	33.6	1	33.6	S29
PVA/MXene sediment	33	1	33	S30
PAAm/CNF/MWCNT	28.5	2	14.25	S31
PAA/chitosan/ACC/RGO	85	9.71	8.75	S32
PAAm/A-11/AgNWs	66	4.1	16.1	S33
PAA/ACC/MXene	45.3	0.13	348.5	S34

Description of Supporting Video

Video S1 The pure shear test demonstration of the NCRO

Supplementary References

[S1] J. Lu, J. Gu, O. Hu, Y. Fu, D. Ye et al., Highly tough, freezing-tolerant, healable and thermoplastic starch/poly (vinyl alcohol) organohydrogels for flexible electronic devices. J. Mater. Chem. A **9**(34), 18406-18420 (2021). https://doi.org/10.1039/D1TA04336F

[S2] Y. Ye, Y. Zhang, Y. Chen, X. Han, F. Jiang, Cellulose nanofibrils enhanced, strong, stretchable, freezing-tolerant ionic conductive organohydrogel for multi-functional sensors. Adv. Funct. Mater. **30**(35), 2003430 (2020). https://doi.org/10.1002/adfm.202003430

[S3] J. Gu, J. Huang, G. Chen, L. Hou, J. Zhang et al., Multifunctional poly (vinyl alcohol) nanocomposite organohydrogel for flexible strain and temperature sensor. ACS Appl. Mater. Interfaces **12**(36), 40815-40827 (2020). https://doi.org/10.1021/acsami.0c12176

[S4] S. Bao, J. Gao, T. Xu, N. Li, W. Chen et al., Anti-freezing and antibacterial conductive organohydrogel co-reinforced by 1D silk nanofibers and 2D graphitic carbon nitride nanosheets as flexible sensor. Chem. Eng. J. **411**, 128470 (2021). https://doi.org/10.1016/j.cej.2021.128470

[S5] L. Xu, D. Qiu, Reversible switching of polymeric gel structure and property by solvent exchange. Sci. China Mater. **65**(2), 547-552 (2022). https://doi.org/10.1007/s40843-021-1824-8

[S6] J. Yang, C. Tang, H. Sun, Z. Liu, Z. Liu et al., Tough, transparent, and anti-freezing

nanocomposite organohydrogels with photochromic properties. ACS Appl. Mater. Interfaces **13**(26), 31180-31192 (2021). https://doi.org/10.1021/acsami.1c07563

[S7] X.-J. Zha, S.-T. Zhang, J.-H. Pu, X. Zhao, K. Ke et al., Nanofibrillar poly(vinyl alcohol) ionic organohydrogels for smart contact lens and human-interactive sensing. ACS Appl. Mater. Interfaces **12**(20), 23514-23522 (2020). https://doi.org/10.1021/acsami.0c06263

[S8] W.-Y. Guo, Q. Yuan, L.-Z. Huang, W. Zhang, D.-D. Li et al., Multifunctional bacterial cellulose-based organohydrogels with long-term environmental stability. J. Colloid Interface Sci. **608**, 820-829 (2022). https://doi.org/10.1016/j.jcis.2021.10.057

[S9] J. Lu, O. Hu, L. Hou, D. Ye, S. Weng et al., Highly tough and ionic conductive starch/poly(vinyl alcohol) hydrogels based on a universal soaking strategy. Int. J. Biol. Macromol. **221**, 1002-1011 (2022). https://doi.org/10.1016/j.ijbiomac.2022.09.083

[S10] M. Li, Y. Yang, C. Yue, Y. Song, M. Manzo et al., Stretchable, sensitive, and environment-tolerant ionic conductive organohydrogel reinforced with cellulose nanofibers for human motion monitoring. Cellulose **29**(3), 1897-1909 (2022). https://doi.org/10.1007/s10570-022-04418-8

[S11] Y. Wu, W. Xing, J. Wen, Z. Wu, Y. Zhang et al., Mixed solvent exchange enabled high-performance polymeric gels. Polymer **267**, 125661 (2023). https://doi.org/10.1016/j.polymer.2022.125661

[S12] D. Wang, J. Zhang, C. Fan, J. Xing, A. Wei et al., A strong, ultrastretchable, antifreezing and high sensitive strain sensor based on ionic conductive fiber reinforced organohydrogel. Composites Part B **243**, 110116 (2022). https://doi.org/10.1016/j.compositesb.2022.110116

[S13] J. Wang, J. Qu, Y. Liu, S. Wang, X. Liu et al., "Crocodile skin" ultra-tough, rapidly self-recoverable, anti-dry, anti-freezing, MoS_2-based ionic organohydrogel as pressure sensors. Colloids Surf., A **625**, 126458 (2021). https://doi.org/10.1016/j.colsurfa.2021.126458

[S14] J. Wang, T. Dai, Y. Zhou, A. Mohamed, G. Yuan et al., Adhesive and high-sensitivity modified $Ti_3C_2T_X$ (MXene)-based organohydrogels with wide work temperature range for wearable sensors. J. Colloid Interface Sci. **613**, 94-102 (2022). https://doi.org/10.1016/j.jcis.2022.01.021

[S15] Y. Zhou, C. Wan, Y. Yang, H. Yang, S. Wang et al., Highly stretchable, elastic, and ionic conductive hydrogel for artificial soft electronics. Adv. Funct. Mater. **29**(1), 1806220 (2019). https://doi.org/10.1002/adfm.201806220

[S16] Q. Rong, W. Lei, L. Chen, Y. Yin, J. Zhou et al., Anti-freezing, conductive self-healing organohydrogels with stable strain-sensitivity at subzero temperatures. Angew. Chem. Int. Ed. **56**(45), 14159-14163 (2017). https://doi.org/10.1002/anie.201708614

[S17] J. Wu, Z. Wu, S. Han, B.-R. Yang, X. Gui et al., Extremely deformable, transparent, and high-performance gas sensor based on ionic conductive hydrogel. ACS Appl.

Mater. Interfaces **11**(2), 2364-2373 (2019). https://doi.org/10.1021/acsami.8b17437

[S18] S. Ko, A. Chhetry, D. Kim, H. Yoon, J. Y. Park, Hysteresis-free double-network hydrogel-based strain sensor for wearable smart bioelectronics. ACS Appl. Mater. Interfaces **14**(27), 31363-31372 (2022). https://doi.org/10.1021/acsami.2c09895

[S19] L. Chen, X. Chang, H. Wang, J. Chen, Y. Zhu, Stretchable and transparent multimodal electronic-skin sensors in detecting strain, temperature, and humidity. Nano Energy **96**, 107077 (2022). https://doi.org/10.1016/j.nanoen.2022.107077

[S20] G. Ge, W. Yuan, W. Zhao, Y. Lu, Y. Zhang et al., Highly stretchable and autonomously healable epidermal sensor based on multi-functional hydrogel frameworks. J. Mater. Chem. A **7**(11), 5949-5956 (2019). https://doi.org/10.1039/C9TA00641A

[S21] J. Lai, H. Zhou, M. Wang, Y. Chen, Z. Jin et al., Recyclable, stretchable and conductive double network hydrogels towards flexible strain sensors. J. Mater. Chem. C **6**(48), 13316-13324 (2018). https://doi.org/10.1039/C8TC04958K

[S22] Z. Wu, H. Ding, K. Tao, Y. Wei, X. Gui et al., Ultrasensitive, stretchable, and fast-response temperature sensors based on hydrogel films for wearable applications. ACS Appl. Mater. Interfaces **13**(18), 21854-21864 (2021). https://doi.org/10.1021/acsami.1c05291

[S23] Q. Wang, Q. Zhang, G. Wang, Y. Wang, X. Ren et al., Muscle-inspired anisotropic hydrogel strain sensors. ACS Appl. Mater. Interfaces **14**(1), 1921-1928 (2022). https://doi.org/10.1021/acsami.1c18758

[S24] Z. Gao, L. Kong, R. Jin, X. Liu, W. Hu et al., Mechanical, adhesive and self-healing ionic liquid hydrogels for electrolytes and flexible strain sensors. J. Mater. Chem. C **8**(32), 11119-11127 (2020). https://doi.org/10.1039/D0TC01094D

[S25] L. Jia, S. Wu, R. Yuan, T. Xiang, S. Zhou, Biomimetic microstructured antifatigue fracture hydrogel sensor for human motion detection with enhanced sensing sensitivity. ACS Appl. Mater. Interfaces **14**(23), 27371-27382 (2022). https://doi.org/10.1021/acsami.2c04614

[S26] C. Fu, Y. Ni, L. Chen, F. Huang, Q. Miao et al., Design of asymmetric-adhesion lignin-reinforced hydrogels based on disulfide bond crosslinking for strain sensing application. Int. J. Biol. Macromol. **212**, 275-282 (2022). https://doi.org/10.1016/j.ijbiomac.2022.05.101

[S27] Z. Zhou, C. Qian, W. Yuan, Self-healing, anti-freezing, adhesive and remoldable hydrogel sensor with ion-liquid metal dual conductivity for biomimetic skin. Compos. Sci. Technol. **203**, 108608 (2021). https://doi.org/10.1016/j.compscitech.2020.108608

[S28] C. Shao, M. Wang, L. Meng, H. Chang, B. Wang et al., Mussel-inspired cellulose nanocomposite tough hydrogels with synergistic self-healing, adhesive, and strain-sensitive properties. Chem. Mater. **30**(9), 3110-3121 (2018). https://doi.org/10.1021/acs.chemmater.8b01172

[S29] Y. Yu, P. Yi, W. Xu, X. Sun, G. Deng et al., Environmentally tough and stretchable

MXene organohydrogel with exceptionally enhanced electromagnetic interference shielding performances. Nano-Micro Lett. **14**(1), 77 (2022). https://doi.org/10.1007/s40820-022-00819-3

[S30] Y. Yang, N. Wu, B. Li, W. Liu, F. Pan et al., Biomimetic porous MXene sediment-based hydrogel for high-performance and multifunctional electromagnetic interference shielding. ACS Nano **16**(9), 15042-15052 (2022). https://doi.org/10.1021/acsnano.2c06164

[S31] W. Yang, B. Shao, T. Liu, Y. Zhang, R. Huang et al., Robust and mechanically and electrically self-healing hydrogel for efficient electromagnetic interference shielding. ACS Appl. Mater. Interfaces **10**(9), 8245-8257 (2018). https://doi.org/10.1021/acsami.7b18700

[S32] D. Lai, X. Chen, G. Wang, X. Xu, Y. Wang, Arbitrarily reshaping and instantaneously self-healing graphene composite hydrogel with molecule polarization-enhanced ultrahigh electromagnetic interference shielding performance. Carbon **188**, 513-522 (2022). https://doi.org/10.1016/j.carbon.2021.12.047

[S33] X. Huang, L. Wang, Z. Shen, J. Ren, G. Chen et al., Super-stretchable and self-healing hydrogel with a three-dimensional silver nanowires network structure for wearable sensor and electromagnetic interference shielding. Chem. Eng. J. **446**, 137136 (2022). https://doi.org/10.1016/j.cej.2022.137136

[S34] Y. Zhu, J. Liu, T. Guo, J. J. Wang, X. Tang et al., Multifunctional $Ti_3C_2T_X$ MXene composite hydrogels with strain sensitivity toward absorption-dominated electromagnetic-interference shielding. ACS Nano **15**(1), 1465-1474 (2021). https://doi.org/10.1021/acsnano.0c08830
